# Spontaneously established syntrophic yeast communities improve bioproduction

**DOI:** 10.1038/s41589-023-01341-2

**Published:** 2023-05-29

**Authors:** Simran Kaur Aulakh, Lara Sellés Vidal, Eric J. South, Huadong Peng, Sreejith Jayasree Varma, Lucia Herrera-Dominguez, Markus Ralser, Rodrigo Ledesma-Amaro

**Affiliations:** 1 Molecular Biology of Metabolism Laboratory, The Francis Crick Institute, London, UK; 2 The Wellcome Centre for Human Genetics, Nuffield Department of Medicine, University of Oxford, Oxford, UK; 3 Department of Bioengineering and Imperial College Centre for Synthetic Biology, Imperial College London, London, UK; 4 Department of Biochemistry, Charité—Universitätsmedizin Berlin, Freie Universität Berlin and Humboldt-Universität zu Berlin, Berlin, Germany; 5 Max Planck Institute for Molecular Genetics, Berlin, Germany

**Keywords:** Metabolic engineering, Synthetic biology

## Abstract

Nutritional codependence (syntrophy) has underexplored potential to improve biotechnological processes by using cooperating cell types. So far, design of yeast syntrophic communities has required extensive genetic manipulation, as the co-inoculation of most eukaryotic microbial auxotrophs does not result in cooperative growth. Here we employ high-throughput phenotypic screening to systematically test pairwise combinations of auxotrophic *Saccharomyces cerevisiae* deletion mutants. Although most coculture pairs do not enter syntrophic growth, we identify 49 pairs that spontaneously form syntrophic, synergistic communities. We characterized the stability and growth dynamics of nine cocultures and demonstrated that a pair of tryptophan auxotrophs grow by exchanging a pathway intermediate rather than end products. We then introduced a malonic semialdehyde biosynthesis pathway split between different pairs of auxotrophs, which resulted in increased production. Our results report the spontaneous formation of stable syntrophy in *S. cerevisiae* auxotrophs and illustrate the biotechnological potential of dividing labor in a cooperating intraspecies community.

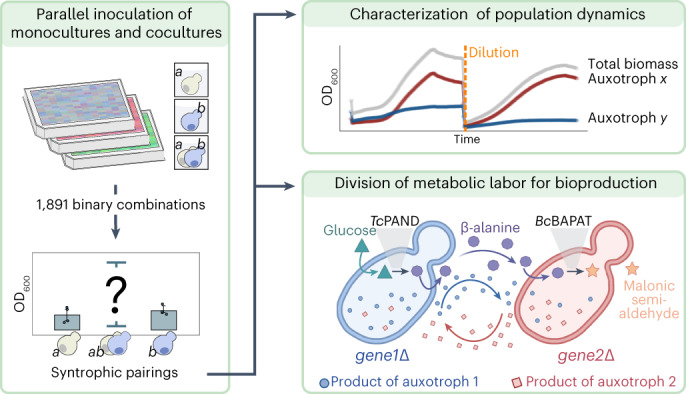

## Abstract (web summary)

Systematically culturing combinations of auxotrophic yeast mutants leads to the identification of pairs that form obligatory cross-feeding relationships, some of which are stable over time and can divide metabolic labor for biotechnological applications.

## Main

An overwhelming majority of microbial species in the wild exist as participants of interspecies and intraspecies communities in which members of microbial communities occupy specific metabolic niches. Microbes often compete, but they can also interact and form cooperative networks that confer adaptive advantages to the communities^1–3^. Irrespective of whether the community members are competing or cooperating, the close proximity of microbes changes the extracellular metabolite environment and results in the exchange of metabolites between cells. It is assumed that the ability to conduct metabolism, not only within but also between cells, can confer extended metabolic capabilities, increases the adaptation potential to fluctuating environments, confers stress resistance and can lead to more efficient metabolic resourcing in challenging growth conditions^3–8^.

One important mechanism of such interactions is obligatory syntrophy—a mutualistic relationship in which two or more organisms survive by feeding off the metabolic products of each other^3,9^. When nearby microbes have complementary metabolic deficiencies, cross-feeding arrangements can form in which the exometabolome of each strain supplies the metabolites required by its neighbor. As our fundamental knowledge of natural microbial communities grows, this well-known characteristic of natural communities becomes increasingly tractable and is, therefore, gaining attention in the field of biotechnology and biomedicine. The ability to manipulate complex microbial interactions could revolutionize the design of genetically engineered biomanufacturing systems, advancing them from single strains to intricate networks that enable new functionalities and improve process efficiencies^10–14^. Indeed, recent work on synthetically engineered intraspecies and interspecies microbial communities has demonstrated the feasibility of dividing metabolic labor by splitting metabolic pathways between subpopulations to improve de novo metabolite synthesis yields, degradation and bioconversion efficiencies and complete heterologous biosynthetic pathways that each member alone is incapable of hosting^13,15–19^. However, it is often challenging to either scale up or maintain stable production due to a reliance on nonsyntrophic genetic circuits designed to maintain subpopulations. These circuits often involve the manual application of chemical inducers or light inputs^20–23^, which can require extensive engineering efforts. In response, recent strategies to maintain the composition of microbial populations have included the use of multiple carbon sources or polymeric microcapsules that physically constrain microbial population ratios^14,24^. In contrast to these strategies, separating metabolic tasks in syntrophic members of a microbial community enables passive, continuous control of subpopulations without the need for multiple growth substrates or physical encapsulation.

However, establishing such stable syntrophic intraspecies or interspecies interactions is not a trivial task. Multiple experimental strategies to enforce metabolic cooperation between auxotrophs have been explored in the past. These include rendering the auxotrophs feedback-resistant, which converts them into metabolite overproducers and improves the growth of complementary auxotrophic pairs that would not otherwise be syntrophic^25^. Such synthetic communities facilitated the separation of biosynthetic modules in time or space^26,27^. However, often the split of a metabolic pathway for biotechnological production requires the exchange of intermediates and not the end products. In contrast to the strategies outlined above, auxotrophic strains that spontaneously enter synergistic interactions without extensive genetic manipulation and optimization would offer a simple and cost-effective method of separating biosynthetic pathways between subpopulations of a single species. While research on this topic has been done in *Escherichia coli*^28,29^, no examples of spontaneously forming intraspecies microbial communities from complementary auxotrophs have been reported thus far in eukaryotes, including *Saccharomyces cerevisiae*, a workhorse in biotechnology.

In fact, the inability *of S. cerevisiae* auxotrophs to cooperate by a simple co-inoculation is a widely accepted general rule^30^. Interestingly, for many auxotrophs, the lack of spontaneous syntrophy cannot be attributed to an insufficient metabolite production or export capacity^31^. Recently, self-establishing metabolically cooperating communities (SeMeCos) were developed that achieve syntrophy between otherwise noncooperative auxotrophs by allowing metabolic interactions to establish via progressive plasmid segregation^31^. The development of the SeMeCo system provided the most concrete evidence obtained so far to support the existence (in nature) and development (in vitro) of metabolically cooperating networks of *S. cerevisiae* mutants that do not require any perturbation of their basal transport capacity or regulatory circuits.

Inspired by this gradually accumulating evidence that *S. cerevisiae* possesses sufficient biosynthetic and metabolite transport capacity for complementing metabolic deficiencies through syntrophic interactions, we hypothesized that there might exist some auxotrophic pairs in yeast that can spontaneously overcome the challenges of establishing a sustainable community. We designed a genome-scale high-throughput screen to test binary combinations of auxotrophs from a prototrophic version of the haploid yeast knockout (YKO) collection for the capacity to exhibit syntrophic growth on synthetic minimal (SM) media^32,33^. Most (97.4%) of the auxotrophic pairs we tested, in concurrence with the established paradigm, did not grow as complementary pairs on a minimal medium. However, we identified 49 pairwise auxotroph combinations formed by 36 unique deletion mutants, for which we observed the spontaneous formation of stable syntrophic interactions upon co-inoculation. A majority (75%) of the successful auxotrophs were deficient in classic amino acid or nucleic acid biosynthesis pathways, while the remaining mapped to protein homeostasis (proteasome, protein maturation, and vacuolar ATPase assembly), transmembrane transport, DNA damage response and the ribosome. We then validated nine auxotroph pairs and characterized their growth characteristics and consortium stability over two consecutive subcultures. Among the highly synergistic, syntrophic communities was a pair of auxotrophs, *trp2∆* and *trp4∆*, bearing deletions in the tryptophan biosynthesis pathway. We characterized this syntrophic interaction and discovered that these mutants cooperate by sharing a biosynthetic intermediate, anthranilate. Finally, for three of these validated and characterized pairs, we introduced a synthetic malonic semialdehyde (MSA) biosynthesis pathway split between the constituent auxotrophs. We demonstrate that syntrophic interactions can be exploited for increasing the production yield of industrially relevant metabolites, by dividing the biosynthesis pathway, and consequently the labor of metabolite synthesis, among two interdependent strains.

## Results

### Few *S. cerevisiae* auxotrophs can form syntrophic communities

**Extended Data Fig. 1:**
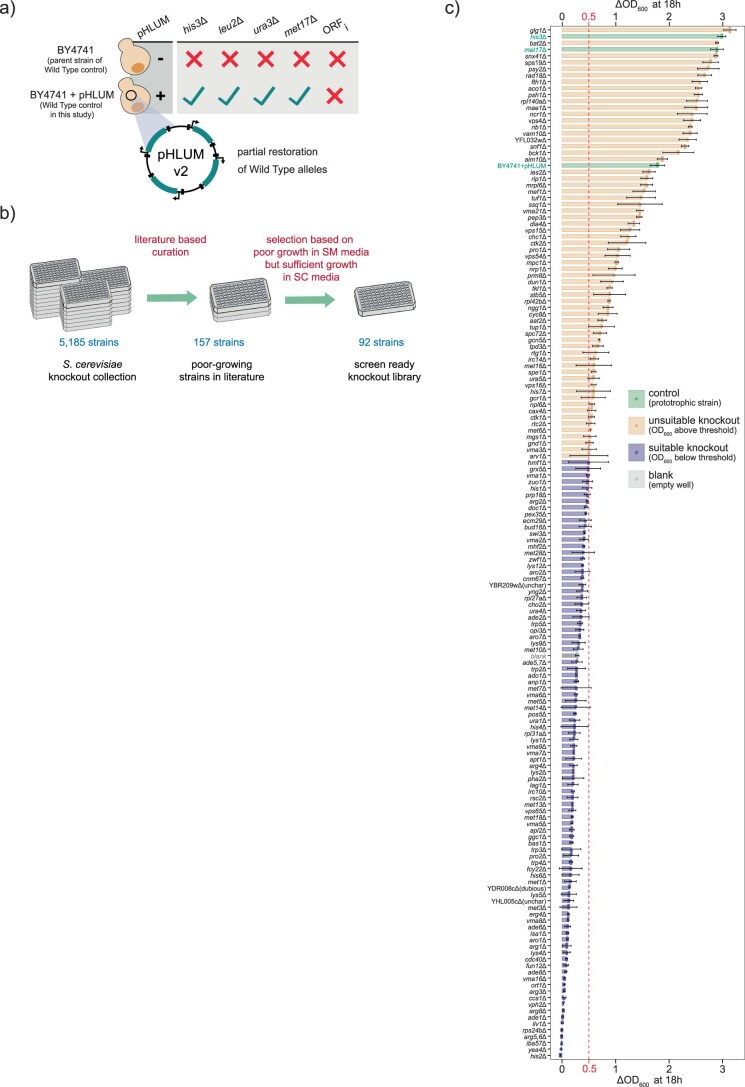
Defining a subset of BY4741-derived deletion strains among the yeast knockout collection. (**a**) BY4741-derived deletion strains were complemented with pHLUM to partially restore wild-type alleles. Since BY4741-derived strains containing pHLUM do not require histidine, leucine, methionine, or uracil to grow in minimal media, any observed growth deficiencies could be linked to other uncomplemented deletion mutations. (**b**) Starting from 5,185 mutants, the library was initially refined to 157, based on previous experimental observations from Mülleder et al.^32^ and then to 92 strains, based on an experiment to measure the growth characteristics of the 157 strains in synthetic minimal media. (**c**) BY4741-derived mutants transformed with pHLUM were cultivated in synthetic minimal media for 18 h. Strains were compared with two known prototrophs: BY4741-derived strains with *met17∆* or *his3∆* deletion mutations (that is, BY4741 mutants with all auxotrophic markers restored by pHLUM and thus no fitness disadvantage; green bars). The red dotted red line represents 20% of average prototrophic growth, which was used as the threshold to choose between mutants that could not grow in synthetic minimal media (purple) and were therefore suitable for the pairwise auxotroph complementation screen and those that were unsuitable (light brown) (see Fig. 1 and Extended Data Figs. 2–5). Error bars (panel c) denote standard error around the mean. Source data

**Extended Data Fig. 2:**
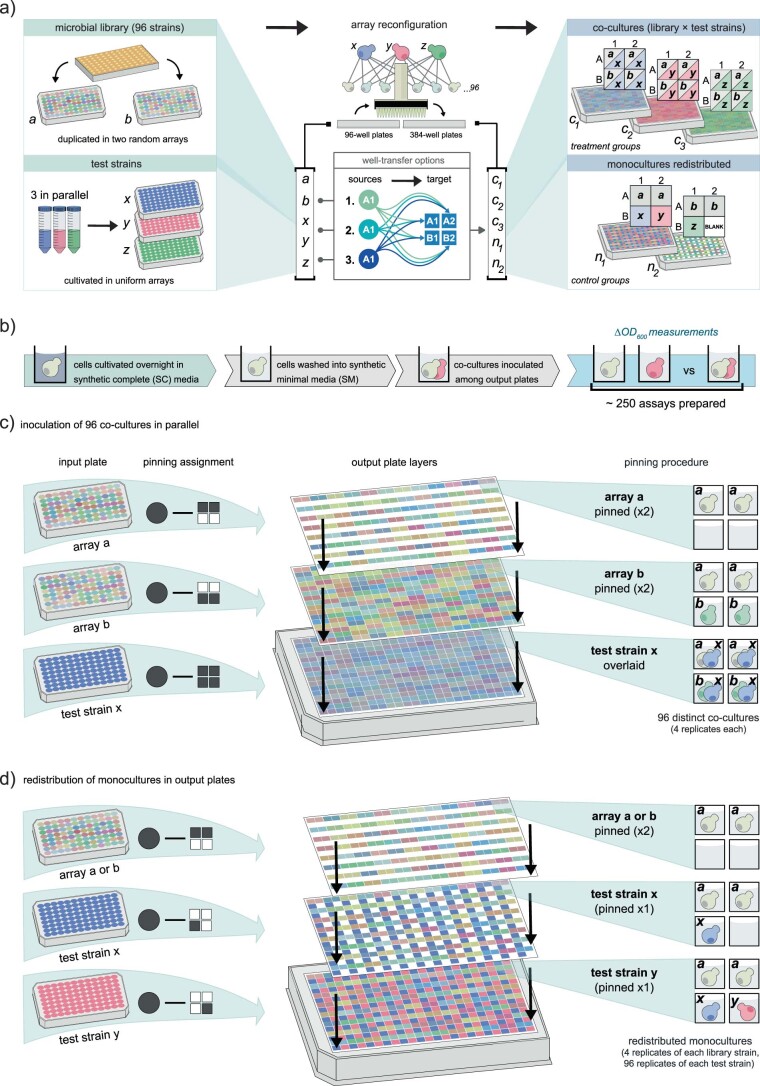
Physical pinning procedure for manoeuvring 96- into 384-well microbial arrays. Growth complementation assays were rapidly prepared in microplates with a robotic pinning arm equipped with disposable plating pads. Relocating cells from 96- to 384-well arrays enabled a stepwise pinning procedure, in which both library and test strains were redistributed as monocultures and combined as co-cultures (**a**) A microbial library of 96 strains (92 auxotrophs + 4 prototrophic controls) was distributed into output plates before test strains were overlaid. Cells within source plates were transferred to target plates through a series of layered pinning operations, where pins would carry ~4 μL of liquid culture and inoculate destination wells (see Supplementary Table 6). Samples had at least 4 replicates, which were either pinned in one output plate (for example, co-cultures) or two different plates (for example, monocultures). (**b**) Strains were cultivated in synthetic complete (SC) to generate biomass for conducting the screen, then washed in synthetic minimal (SM) media before inoculation in SM media for the auxotroph complementation screen. (**c**) Visual depiction of the experimental procedure of preparing co-culture layouts. (**d**) Visual depiction of the experimental procedure of preparing monoculture layouts.

**Extended Data Fig. 3:**
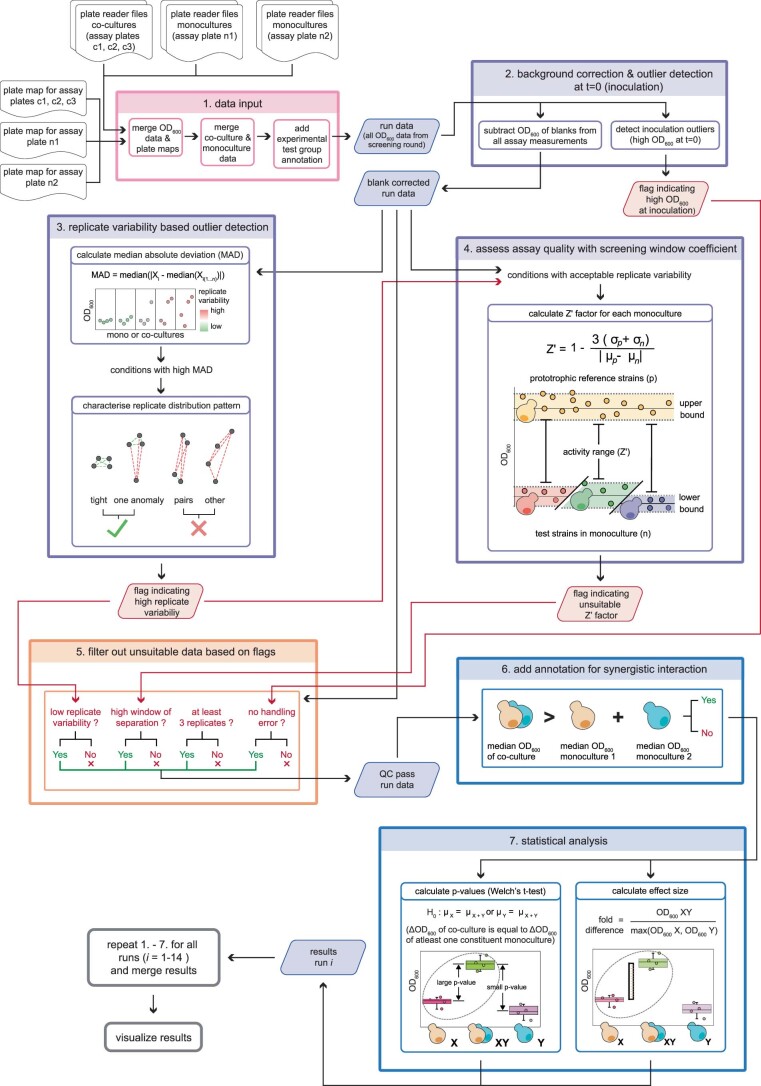
Analytical pipeline for processing growth complementation assays. Statistical thresholds and conditional statements were designed to parse OD_600_ datasets, conduct quality assessments, and categorise co-cultures by their complementary behaviour (Supplementary Note). Co-cultures were issued annotations and quality metrics which guided hit selection. The pipeline comprises 7 steps: 1) Data input and annotation. 2) Background correction and outlier detection. 3) detection of spatial bias and inconsistent growth amongst biological replicates. 4) Assessing the quality of each assay using the screening window coefficient (Z’ factor). 5) Removing unsuitable auxotrophs and growth complementation assays based on assays deemed unsuitable in 2), 3) and 4). 6) Annotating co-cultures for the presence of synergistic interactions and 7) Statistical analysis.

**Extended Data Fig. 4:**
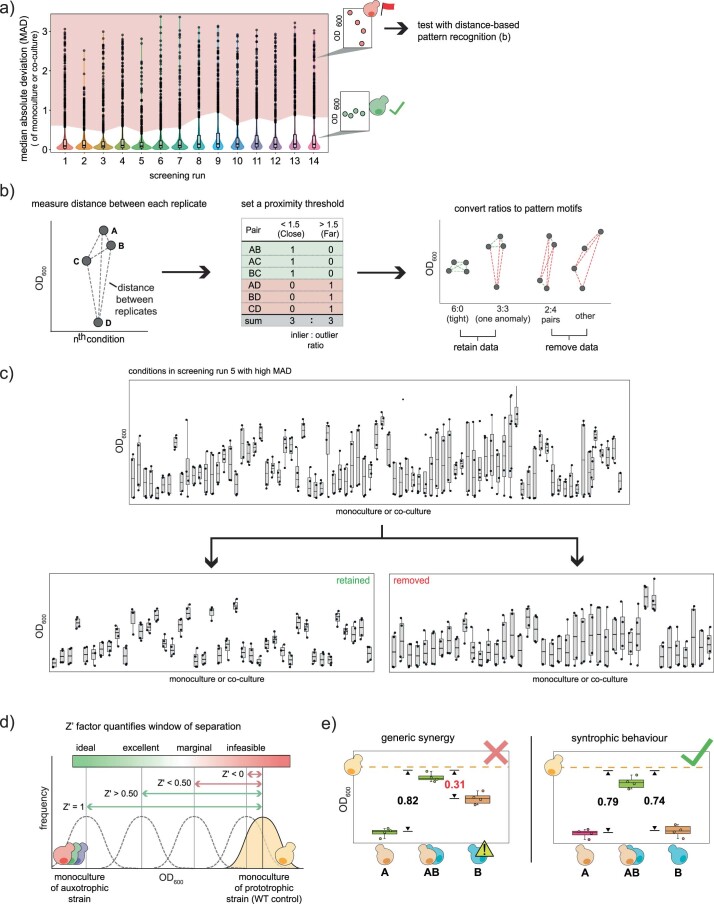
Removing samples with inconsistent growth between replicates. (**a**) Median absolute deviations calculated for each monoculture and co-culture across all 14 screening runs. Outlying MAD values (values beyond Q3 + 1.5×IQR) were subject to a distance-based classification algorithm for pattern recognition. (**b**) The distance-based pattern recognition algorithm helps locate assay-specific spatial bias by classifying replicate distributions, in which distances (defined as the difference in OD_600_) were calculated between all replicate points. Culture conditions received a mix of ‘far’ or ‘close’ labels, which were then summarised into an ‘inlier-outlier’ ratio. These ratios translated into specific univariate patterns, and thus enabled pattern recognition of replicate distributions. (**c**) An example of workflow described in (a) and (b) Culture conditions with high replicate variance from screening run 5 were categorised by their inlier-outlier ratios. Instead of discarding all conditions with high replicate variance, the written algorithm enables a refined approach for selective data discarding, minimising the premature removal of assays from the pipeline that could lead to the identification of real syntrophic communities (Supplementary Note 1). (**d**) Quality assessments to isolate auxotrophs from experimental screen data. The Z’-Factor is a measure of effect size, which is a dimensionless statistic commonly used to evaluate assay quality in high-throughput screening^34^. Variance and dynamic range were measured among test strains and prototrophs (producing values ranging around −1 and 1). Each growth complementation assay had a unique activity range, which was defined by both prototrophs (positive controls) and auxotrophs (negative controls). (**e**) Monocultures that earned a Z’-Factor of 0.50–1.00 were considered excellent quality, while coefficients less than 0.50 were interpreted as inadequate (that is, as having too much signal overlap between negative and positive controls). Assay quality (that is, separation between lower and upper bounds) was directly impacted by monoculture growth. Isolating strains with a Z’-Factor above 0.50 selected for both high-quality assays and low-growing monocultures. Error bars (panels a, c, and e) denote standard deviation around the mean.

To identify auxotrophies, we used an *S. cerevisiae* gene-deletion library comprised of 5,185 knockout mutants harboring the pHLUM minichromosome to complement the four auxotrophies (*his3∆, leu2∆, met15∆,* and *ura3∆*; Extended Data Fig. 1a) of the parent BY4741 strain^32^ and compared their growth in nutrient-supplemented synthetic complete (SC) and on SM media, which lacks amino acid and nucleotide supplements (Methods). Ninety-two strains showed poor growth (defined as 20% of the parent strain’s optical density at 600 nm (OD_600_)) after 18 h in SM, but grew well on SC (Extended Data Fig. 1c). A total of 73% of these strains contained gene deletions directly involved in amino acid or nucleotide biosynthesis pathways. To test if any of these auxotrophs could form a syntrophic community with a complementary strain, every auxotroph was inoculated with each of the other 91 in liquid SM media in a high-throughput manner using automated colony-picking and liquid-handling robots (Extended Data Fig. 2). Cell density (OD_600_) in each well was recorded after 48 h. Quality control filters excluded samples showing inconsistent growth patterns and possible contamination. A total of 62 monocultures (Supplementary Table 1) and 1,891 cocultures (Supplementary Data 1) passed the quality control checks. Synergistic and syntrophic growth was detected by combining a Z-factor metric^34^ with the growth advantage of a community over the individual growth of its most successful constituent auxotroph, *P* values from Welch’s *t*-test (corrected for multiple testing using the Benjamini–Hochberg method) and fold difference in OD_600_ relative to the auxotroph with higher growth among the pair in SM (Methods, Supplementary Note and Extended Data Figs. 3 and 4).

**Fig. 1:**
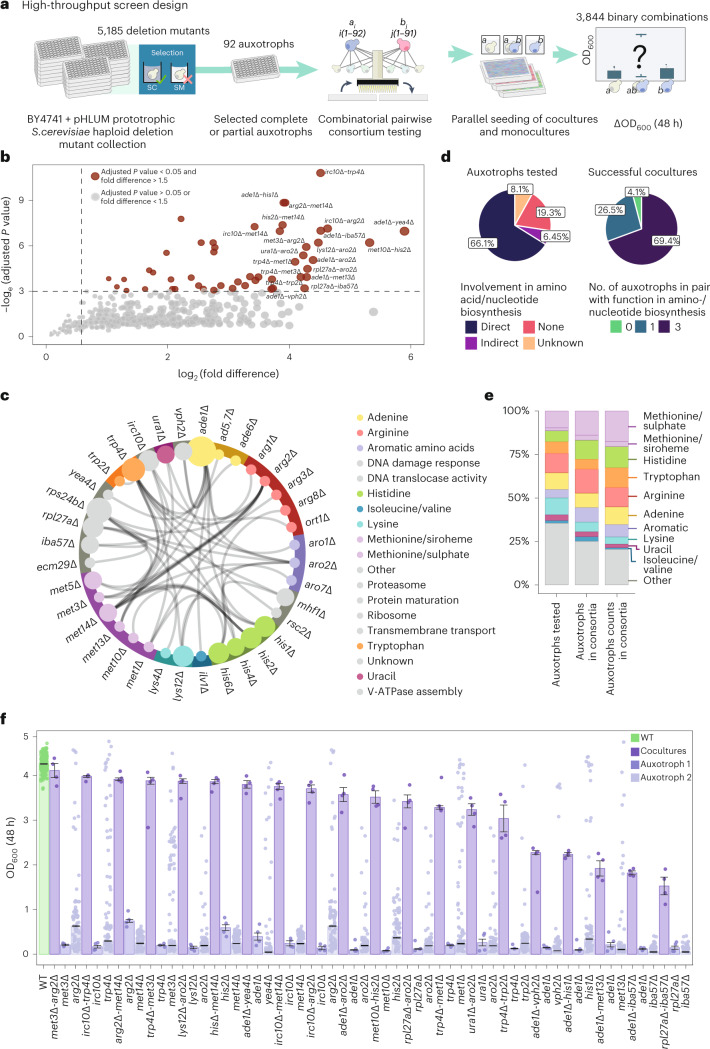
High-throughput growth complementation screen overview. **a**, Summary of the physical workflow for discovering auxotroph pairs capable of syntrophic and synergistic growth. Ninety-two BY4741-derived strains harboring the pHLUM plasmid were selected for growth complementation assays. Coculturing auxotrophs in minimal media imposes a ‘sink or swim’ scenario, in which neighboring cell populations must spontaneously cooperate by cross-feeding for collective growth. Our study sought to discover yeast strains that grew better in mixed cultures than in corresponding pure cultures. **b**, *P* value threshold of less than 0.05 from a two-sided Welch’s *t*-test and fold difference threshold of 1.5 calculated out between the fittest constitute auxotroph and coculture for each complementation assay was used to select cocultures that showed substantially better growth than any constituent monoculture. *X* axis, log_2_(OD_600_ coculture/OD_600_ fittest constituent auxotroph); *y* axis, log_e_ (*P* value of Welch’s *t*-test after correction for multiple testing using the Benjamini–Hochberg method). **c**, All pairs of statistically significant synergistic cocultures discovered in the study. Colors indicate molecular function curated using the Gene Ontology database. **d**, Pie charts indicating the enrichment of auxotrophs belonging to amino acid and nucleotide biosynthesis pathways in successful cocultures when compared to all auxotrophs that were tested. **e**, Distribution of auxotrophs belonging to the top 10 most enriched pathways across each stage of our screen (all auxotrophs tested, unique gene deletions present in the successful cocultures and counts of each gene deletion in the successful cocultures). **f**, Bar plots representing OD_600_ of the top 20 most successful cocultures from growth complementation assays. Cocultures and their constituent auxotroph monocultures are ordered by the fold difference of the coculture versus the fittest monoculture in decreasing order. WT refers to the prototrophic BY4741 + pHLUM monoculture. Samples had at least *n* = 4 biological replicates. Some monocultures had *n* = 96 biological replicates, due to a logistical constraint in our automated pinning procedure, which was designed to consume minimal plastic plating pads while generating all target plate conditions (Extended Data Fig. 2 and Supplementary Table 6). Error bars in **f** denote standard error around the mean. Source data

**Extended Data Fig. 5:**
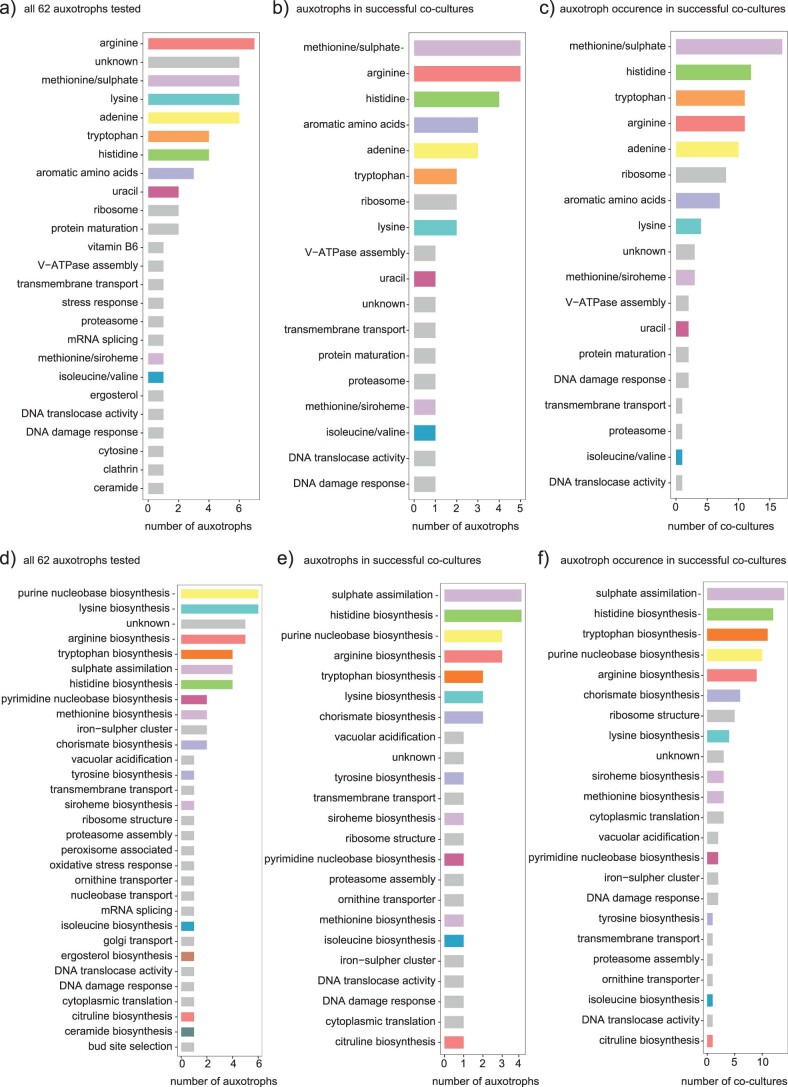
Metabolic products associated with cell auxotrophies. (**a**) Relative distribution of metabolic products associated with the 62 auxotrophs that passed quality control checks. (**b**) All gene deletions present in the 49 successful co-cultures. (**c**) Gene deletions weighted by their number of occurrences in any of the 49 co-cultures (that is, accounting for promiscuous auxotrophs that formed communities with multiple partners). (**d**) Relative distribution of gene ontology terms associated with the 62 auxotrophs that passed quality control checks. (**e**) All gene deletions present in the 49 successful co-cultures. (**f**) Gene deletions weighted by their number of occurrences in any of the 49 co-cultures (that is, accounting for promiscuous auxotrophs that formed communities with multiple partners). Source data

In total, 1,842 of 1,891 (97.4%) auxotrophic pairs tested were unable to grow in SM. However, 36 unique gene deletions in different pairwise combinations of a total of 49 cocultures (2.6%) were found to grow substantially better than each of the corresponding auxotrophs individually (Fig. 1b,c and Supplementary Table 2; raw and processed data for all strains in Supplementary Data 2–4). Most (96% or 47/49 pairs) of these successful cocultures contained at least one strain in which the deleted gene has a known functional association to amino acid or nucleotide biosynthesis (Fig. 1d and Supplementary Table 2) and 75% (27/36) of the unique gene deletions encode enzymes that directly participate in amino acid or nucleotide biosynthesis. Thus, in our screen, we primarily detect the capacity of auxotrophs bearing direct enzyme deletions to form spontaneous, syntrophic communities (Supplementary Table 1). In total, 89% of the auxotrophs were associated with deletions in just the following nine metabolic pathways: methionine and organic sulfur cycle, histidine, tryptophan, arginine, adenine, lysine, uracil, isoleucine/valine and the aromatic amino acid superpathway (Fig. 1e, Extended Data Fig. 5 and Supplementary Table 1). It should be noted, however, that this list of pathways is influenced by the nature of the growth medium used^35^, which contains several trace elements and vitamins. Other culture conditions, such as growth in more minimal media, may uncover additional auxotroph pairs (with alternative pathway deletions) capable of establishing cooperative relationships.

**Extended Data Fig. 6:**
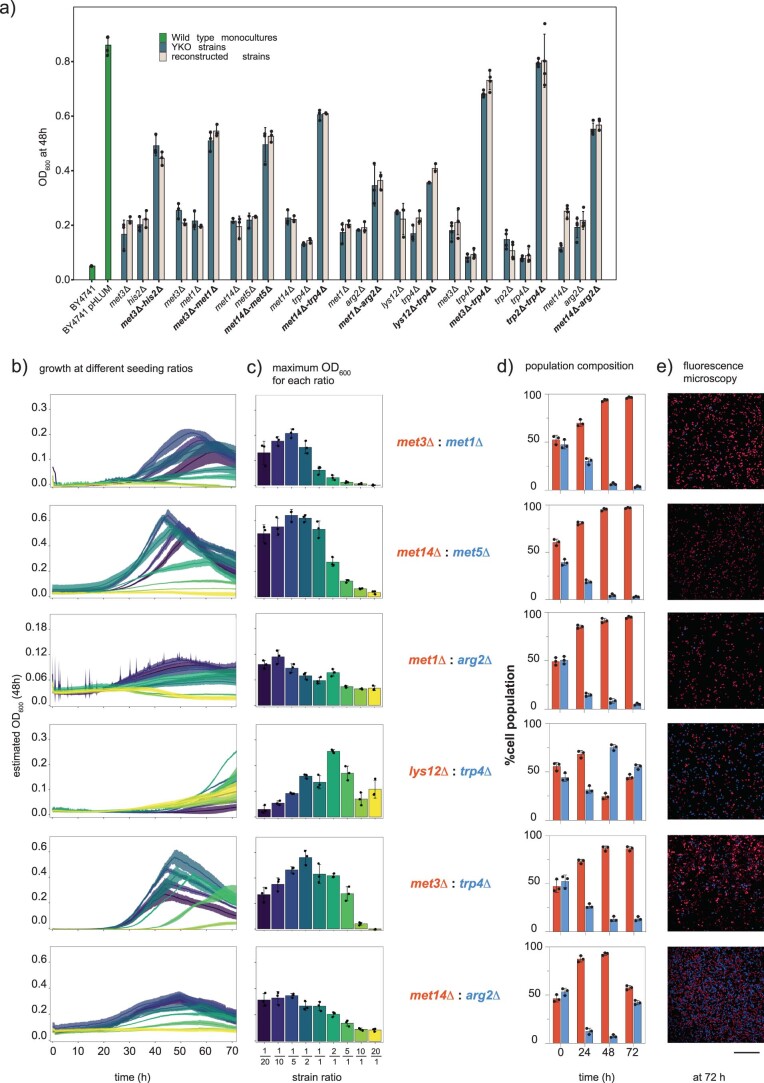
Validating auxotrophic pairs with growth complementation assays. (**a**) Synergistic growth phenotypes were compared between strains from the primary screen collection and freshy-created auxotrophs (that is, deletion mutants) constructed from a separate BY4741 lineage. Pairs of auxotrophs re-established a synergistic community by simple co-inoculation, indicating a high agreement between the YKO (BY4741 + pHLUM knock-out mutation) library and independently generated knock-out strains. Error bars denote standard deviation around the mean. of 3 or 4 biologically independent experiments. (**b**) Characterising growth behaviour of six additional reconstituted syntrophic yeast co-cultures overtime. Co-cultures were inoculated at 9 different inoculation ratios (that is, different proportions of pairwise auxotrophs), and then OD_600_ was measured overtime. (**c**) Success of growth complementation in syntrophic yeast consortia was sensitive to population densities and ratios upon initial inoculation. (**d**) In order to characterise population dynamics, the constituent auxotrophs of each co-culture were tagged with either BFP or mScarlet. Strain proportions within each population were estimated by measuring blue and red fluorescence over a period of 72 h. (**e**) Fluorescence micrographs of blue- and red- fluorescing syntrophic co-cultures. Co-cultures were cultivated in minimal media, extracted at 72 h, fixed with paraformaldehyde, and then transferred to microplates for fluorescence imaging. Micrograph results were comparable to those obtained by measuring fluorescence at the population level. Shaded area (panel b) or error bars (panels a, c and d) denote standard deviation around the mean of 3 biologically independent experiments. The scale bar corresponds to a length of 100 μm. Source data

Because deletion mutant libraries are known to be susceptible to problems such as accumulation of secondary mutations during passaging and occasional cross-contamination, we validated our results by reconstructing a subset of auxotrophs by introducing deletions de novo in the BY4741 parental strain by homologous recombination. Among the top 49 cocultures in our screen, we found a pair of successful auxotrophs (*trp4*Δ–*trp2*Δ) that mapped to the same biosynthetic pathway, suggesting that syntrophic interactions could form through the exchange of pathway intermediates. Therefore, we revisited our screen data, looking for auxotroph pairs within single metabolic pathways. For instance, the methionine auxotrophies are known to be leaky because cells can share intermediates of the organic sulfur cycle, such as sulfide ions, to support growth^36,37^. We reconstrued two cocultures (*met3*Δ–*met1*Δ and *met14*Δ–*met5*Δ) that were at the significance threshold of our screen (adjusted *P* values were just above the 0.05 threshold, and fold change of the OD_600_ coculture in comparison to the OD_600_ of the fittest monoculture was high; Supplementary Data 4). Of the eleven auxotroph pairs that we recreated from fresh deletion mutants, nine re-established a synergistic, syntrophic community by simple co-inoculation, indicating a high agreement between the gene-deletion library and independently and freshly generated knockout strains (Supplementary Table 3 and Extended Data Fig. 6a).

### Characterizing growth dynamics of syntrophic cocultures

**Fig. 2:**
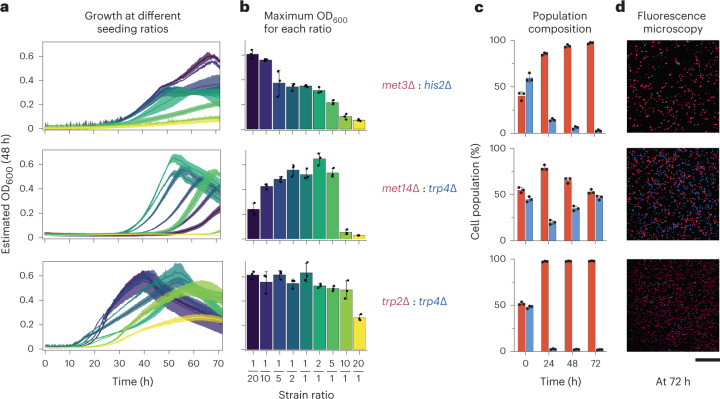
Characterizing growth behavior of three reconstituted syntrophic yeast cocultures over time. **a**, Cocultures were inoculated at nine different inoculation ratios (that is, different proportions of pairwise auxotrophs), and then OD_600_ was measured over time. **b**, Success of growth complementation in syntrophic yeast consortia was sensitive to population densities and ratios upon initial inoculation. **c**, To characterize population dynamics, auxotrophs involved in each coculture were tagged with either BFP or mScarlet. Strain proportions within each population were estimated by measuring blue and red fluorescence over a period of 72 h. **d**, Fluorescence micrographs of blue- and red-fluorescing syntrophic cocultures. Cocultures were cultivated in minimal media, extracted at 72 h, fixed with paraformaldehyde and then transferred to microplates for fluorescence imaging. Micrograph results were comparable to those obtained by measuring fluorescence at the population level. Shaded area (**a**) or error bars (**b**,**c**) denote standard deviation around the mean of *n* = 3 independent biological replicates. The scale bar corresponds to a length of 100 μm. Source data

We next characterized population dynamics and interactions of nine validated cocultures by tagging the constituent auxotrophs in each pair with a fluorescence protein, either blue fluorescent protein (BFP) or mScarlet, and then estimating the proportions of intermixed populations over time using fluorescence readouts from a spectrophotometer as well as fluorescence microscopy (Fig. 2). Cultivation success in syntrophic yeast consortia was sensitive to population densities and proportions. Changes to inoculation ratios had a considerable impact on both the duration of the lag phase and maximal growth (Fig. 2a,b). Each consortium had an optimal inoculation ratio in which the lag phase was the shortest and maximal growth the highest, which suggests that co-auxotrophic strains require specific extracellular conditions that must be satisfied before entry into exponential growth. Prolonged lag phases may correspond to a required ‘greeting’ period, in which complementary auxotrophs in syntrophic populations must reciprocally adapt their metabolic networks such that the export of supplies from each auxotroph meets the import demands of its partner. We then inoculated cocultures in a 1:1 ratio and tracked with fluorescence microscopy how communities migrated toward a defined population ratio over time (Fig. 2c,d). Population analysis revealed different growth patterns among the tested cocultures, which can be broadly grouped into the following two categories: equally balanced growth of both strains (such as for *met14*Δ*–trp4*Δ and *met14*Δ–*arg2*Δ), and clear predominance of one strain (for example, in the cases of *met14*Δ–*met5*Δ and *trp2*Δ–*trp4*Δ; Fig. 2c). The different dynamics of the tested cocultures can be attributed to multiple factors, such as the rate of diffusion of the shared metabolites and the rate of influx required by each auxotrophic strain of a given metabolite to sustain growth. The fact that a variety of population ratios could support consortium growth indicates that syntrophic relationships are flexible, emergent mechanisms that adapt to the requirements imposed by the metabolic capabilities of the strain involved^38^.

**Fig. 3:**
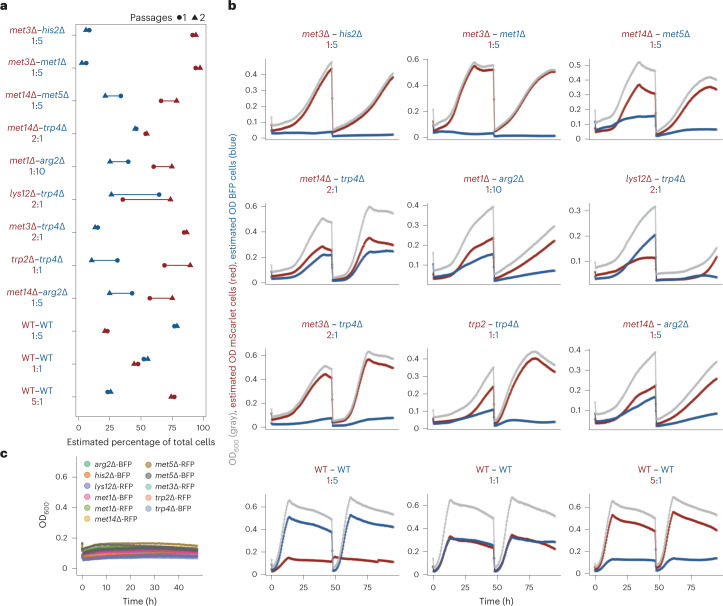
Population ratios in each coculture persist across serial dilutions. Cocultures were tagged with either BFP or mScarlet, inoculated at different inoculation ratios, cultivated for 48 h and then diluted (to OD_600 of 0.10_) into SM media. **a**, Strain ratios of syntrophic cocultures evolved with a similar trend as that observed before dilution. **b**, Syntrophic cocultures display subpopulation drift during exponential growth. WT (BY4741 + pHLUM) cocultures were also inoculated at different ratios, where WT variants were either tagged with BFP or mScarlet. WT cocultures showed no subpopulation drift during exponential growth. **c**, Auxotroph monocultures (negative controls) grow poorly in SM media. Source data

**Extended Data Fig. 7:**
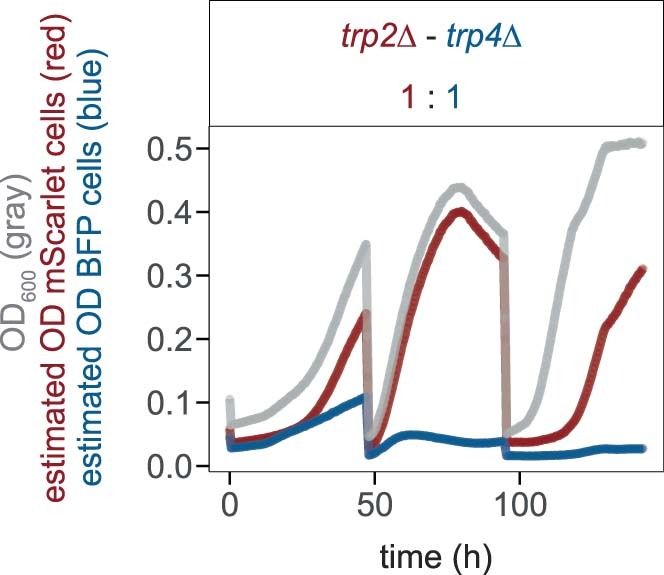
The *trp2∆-trp4∆* co-culture is stable after repeated re-inoculations and dilutions despite extreme imbalance of auxotroph ratios. *trp2∆-trp4∆* cultures were serially cultivated for 48 h and then washed and diluted (to OD_600_ of 0.10) into SM. In general, when co-cultures were reinoculated into minimal medium, strain ratios evolved with a similar trend as that observed before dilution, and the overall cell density increased in a manner like that observed during the first cultivation period. Source data

Because the stability of a coculture is critical for its use in industrial processes, we next tested whether the coculture and its distinct population ratios would re-emerge upon serial dilutions. Cell cultures were grown for 48 h and then washed and diluted (to OD_600_ of 0.10) into SM (Fig. 3). In general, when cocultures were re-inoculated into a minimal medium, strain ratios evolved with a similar trend as that observed before dilution (except for *lys12∆–trp4∆* coculture), and the overall cell density increased in a manner similar to that observed during the first cultivation period. Instances where re-inoculated cocultures achieve comparable or increased levels of growth, despite diverging from the previously defined optimal ratios, may be due to adaptation and co-evolution of the community over time. Still, in 6 of 9 cocultures, we find the expected behavior of slower growth after re-inoculation at nonoptimal ratios, which indicates that if an adaptation occurs, this would be coculture or amino acid dependent. Together, this experiment demonstrates that consortia-dependent interactions can be stable and that both strains in each consortium are viable by the end of batch culture (even for pairs with an extreme imbalance of auxotroph ratios, such as the *trp2*Δ*–trp4*Δ pair) as they are able to regrow syntrophically upon re-inoculation (Supplementary Data 5 and Extended Data Fig. 7).

### *trp2*∆–*trp4*∆ exchanges both intermediates and end products

**Fig. 4:**
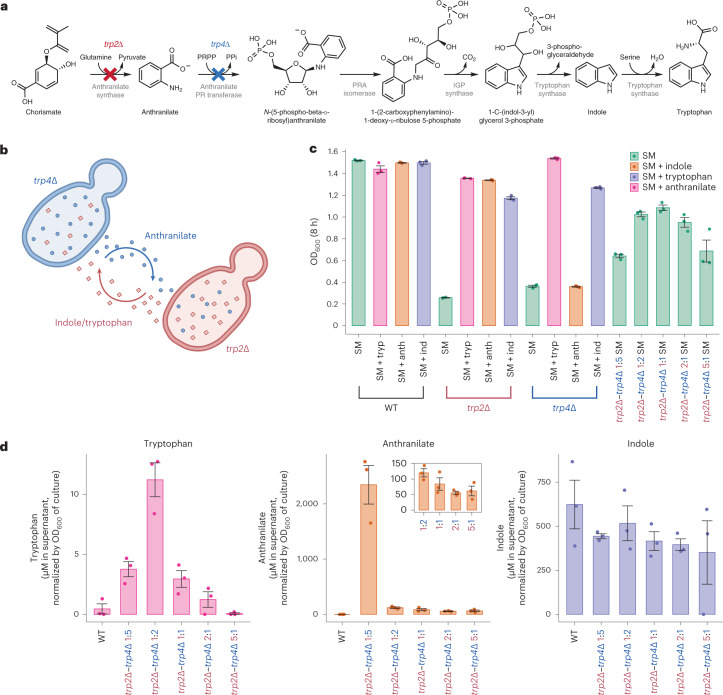
Characterizing a *trp2*∆–*trp4*∆ syntrophic community. **a**, The *S. cerevisiae* tryptophan biosynthesis pathway. Reactions catalyzed by enzymes deleted in *trp2∆* and *trp4∆* strains are marked with red and blue crosses, respectively. **b**, Diagram of probable metabolites being exchanged by the *trp2∆*–*trp4∆* community. Between chorismate and tryptophan, three of the intermediate metabolites (phosphoribosyl-anthranilate, carboxyphenyl amino-deoxyribose-5-phosphate and indole-3-glycerol phosphate) are phosphorylated (which render them less likely to cross the cell membrane^40^). **c**, OD_600_ after 8 h of cultivation of each monoculture (*trp2*Δ, *trp4*Δ and WT) and coculture (*trp2*Δ–*trp4*Δ) in either SM liquid media, SM supplemented with tryptophan, SM supplemented with anthranilate, or SM supplemented with indole. **d**, Metabolite concentrations of tryptophan, anthranilate and indole across the *trp2Δ*–*trp4Δ* coculture when inoculated at different inoculation ratios. Error bars (**c**,**d**) denote standard error around the mean. Source data

The main potential of syntrophic communities’ biotechnology would be to split the metabolic burden of a biosynthetic pathway between multiple cells. This would, however, typically not entail the exchange of the pathway’s end products, but of the intermediates. Interestingly, among our validated synergistic communities, we identified two cases that seemed to be explained by the exchange of intermediates. In one case, these involve methionine auxotrophs deficient for the organic sulfur cycle. As we and others have shown recently, organic sulfur auxotrophy can be overcome by the fixation of inorganic sulfur (that is, sulfide ions) that leak upon the perturbation of the methionine pathway^36,39^. Moreover, we identified a pair of auxotrophs (*trp2∆* and *trp4∆*) that lack subsequent enzymes in the tryptophan biosynthesis pathway. The structure of the metabolic pathway implied that *trp2∆* and *trp4∆* strains would have to share at least one biosynthetic intermediate rather than an inorganic ion, most likely anthranilate, which is product of Trp2p and substrate of Trp4p, in addition to either the end product tryptophan or one of the four intermediates between anthranilate and tryptophan (Fig. 4a,b). Because three of the intermediate metabolites (phosphoribosyl-anthranilate, carboxyphenyl amino-deoxyribose-5-phosphate and indole-3-glycerol phosphate) are relatively unstable, phosphorylated metabolites that are unlikely to readily cross the cell membrane^40^, we quantified anthranilate, indole and tryptophan.

**Extended Data Fig. 8:**
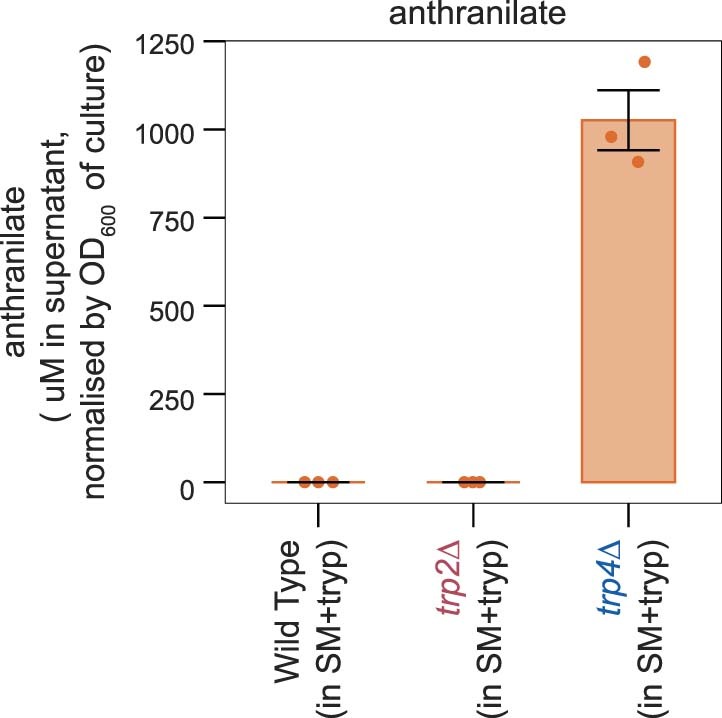
Anthranilate was detected in the supernatant of *trp4*∆ monocultures but not in WT or *trp2*∆ monocultures. Concentration of anthranilate as quantified by LC-MS in the supernatant of each monoculture (WT; BY4741 + pHLUM), *trp2∆* and *trp4∆* auxotrophs) cultivated in SM + tryptophan for 8 h. Y axis: anthranilate (in µM) normalised by OD_600_ of sampled monoculture. Error bars denote standard error around the mean. Source data

To measure the concentrations of anthranilate, indole and tryptophan in the culture medium of the syntrophic *trp2∆*–*trp4∆* community, each auxotrophic monoculture and the prototrophic parental strain (BY4741-pHLUM (WT)) were measured using a targeted liquid chromatography–mass spectrometry (LC–MS) assay after 8 h of growth (Methods; Supplementary Table 4). We detected a large increase in the extracellular concentration of anthranilate in the *trp2∆–trp4∆* community in SM as well as *trp4∆* in SM supplemented with tryptophan (SM + tryp) but could not detect anthranilate in WT or *trp2∆* (Extended Data Fig. 8 and Supplementary Data 6). The extracellular concentration of anthranilate was proportional to the fraction of *trp4∆* cells in the inoculum of the community (Fig. 4d, middle panel, and Supplementary Data 6). A slightly different pattern was observed for tryptophan, with the *trp2∆*–*trp4∆* community inoculated at a 1:2 ratio exhibiting the maximum extracellular tryptophan concentration (Fig. 4d, left panel, and Supplementary Data 6). No significant differences in indole concentration between the WT and any of the cocultures were observed, which could indicate that the export and consumption rate is very similar, the amount exchanged is a small fraction of the indole secreted or that it is not exchanged.

To further corroborate our observation that the biosynthetic intermediate, anthranilate, is exchanged between the two auxotrophs, we inoculated *trp2∆* and *trp4∆* cells in SM supplemented with anthranilate. Consistent with our hypothesis, the addition of anthranilate to SM media restored the growth of *trp2∆* but not *trp4∆*, while the addition of tryptophan restored the growth of both strains (Fig. 4c and Supplementary Table 5). Finally, as would be expected because indole is downstream of the reactions catalyzed by Trp2p and Trp4p, the addition of indole to SM partially rescued the growth of both *trp2∆* and *trp4∆* (Fig. 4c). These observations suggest that this coculture exchanges anthranilate and either tryptophan or indole or both.

### Division of labor increases MSA production

**Fig. 5:**
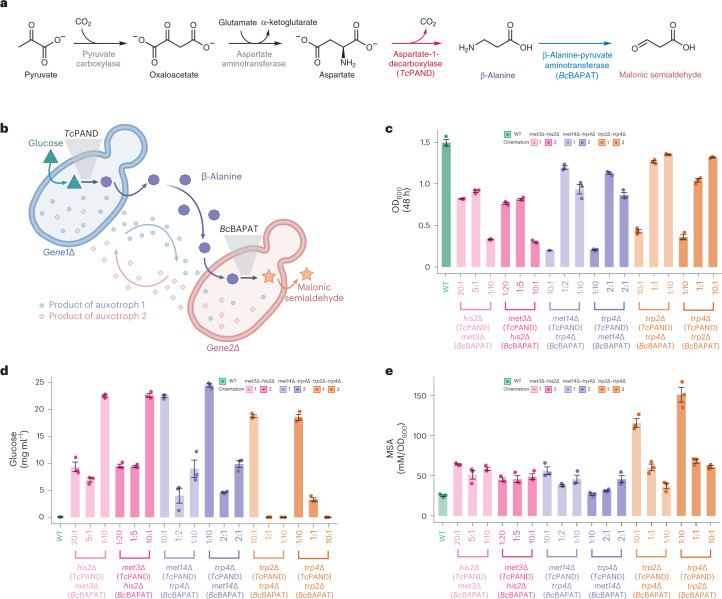
Comparing MSA bioproduction across different syntrophic cocultures. **a**, Metabolic pathway for the production of MSA from pyruvate is composed of four enzymes (two are natively present in *S. cerevisiae* (pyruvate carboxylase and aspartate aminotransferase) and two are exogenous enzymes (*T. castaneum* aspartate decarboxylase (*Tc*PAND) and *B. cereus* β-alanine-pyruvate aminotransferase (*Bc*BAPAT)). **b**, Diagram of the genetically engineered coculture that represents both the cross-fed metabolites being exchanged by the syntrophic cocultures and the export/uptake of β-alanine as part of the heterologous MSA heterologous pathway. **c**, Division of metabolic labor increases the efficiency of the MSA pathway when split between syntrophic cocultures (*his2Δ*–*met34Δ*, *met14Δ*–*trp4Δ* and *trp2Δ*–*trp4Δ*). Growth and production varied with changes in the inoculation ratios for all cocultures. The orientation of the MSA biosynthesis (that is, which auxotroph carried each exogenous enzyme) also had a minor impact on the production titer. OD_600_ (**c**) and glucose concentration (**d**) in the cultivation media for each coculture at 48 h. **e**, When the MSA production titer was normalized by OD_600_ of each coculture at least one starting inoculation ratio of all cocultures outperformed the monoculture. Error bars (**c**,**d**) denote standard error around the mean. Source data

The cocultures discovered and validated in this study provide a stable, spontaneously establishing system comprising two mutants of the same species, that can, in principle, be exploited to enforce a division of the costs to sustain a heterologous metabolic pathway introduced into each mutant. In addition, the varied composition of subpopulations in each of the nine presented cocultures serves as an additional feature, which can be changed alongside metabolic pathway-specific parameters to tune carbon distributions, growth and overall biomanufacturing performance in the microbial community. Therefore, we next tested whether a division of metabolic labor would increase the efficiency of a metabolic pathway of biotechnological interest split between syntrophic pairs. As a test case, we aimed to improve the production of MSA, a precursor metabolite useful for a variety of industrial purposes, such as the production of biodegradable polymers^41^. For this purpose, we chose a previously established synthetic pathway that comprises the following two core enzymes: aspartate-1-decarboxylase from *Tribolium* *castaneum* (*Tc*PAND, encoded by the *LOC100124592* gene) and β-alanine-pyruvate aminotransferase from *Bacillus cereus* (*Bc*BAPAT, encoded by the *yhxA* gene)^42^ (Fig. 5a).

First, we tested how the division of labor in nonsyntrophic cocultures performed in comparison to a monoculture. For that, we created three control strains, one as the WT monoculture and two to form the WT coculture. The WT monoculture bearing both enzymes in a single strain was used as a control for bioproduction without any division of labor, while the WT coculture, formed by two strains (one expressing the gene encoding *Tc*PAND and the other expressing the gene encoding *Bc*BAPAT), was used as a control for division of labor without any enforced syntrophy (and consequent lack of control of the subpopulation ratios). First, we compared the growth and production titers of the WT monoculture with the WT coculture inoculated at different ratios (1:10, 1:1 and 10:1). We observed that the OD_600_ of the monoculture was lower, which indicates a higher metabolic burden of expressing the two genes in the same cell. However, the production of MSA was also higher in the monoculture, suggesting a better conversion of the carbon source into the product (Supplementary Data 7). These results indicate that division of labor may reduce metabolic burden, but that is not enough to improve production over the monoculture in a nonsyntrophic scenario.

**Extended Data Fig. 9:**
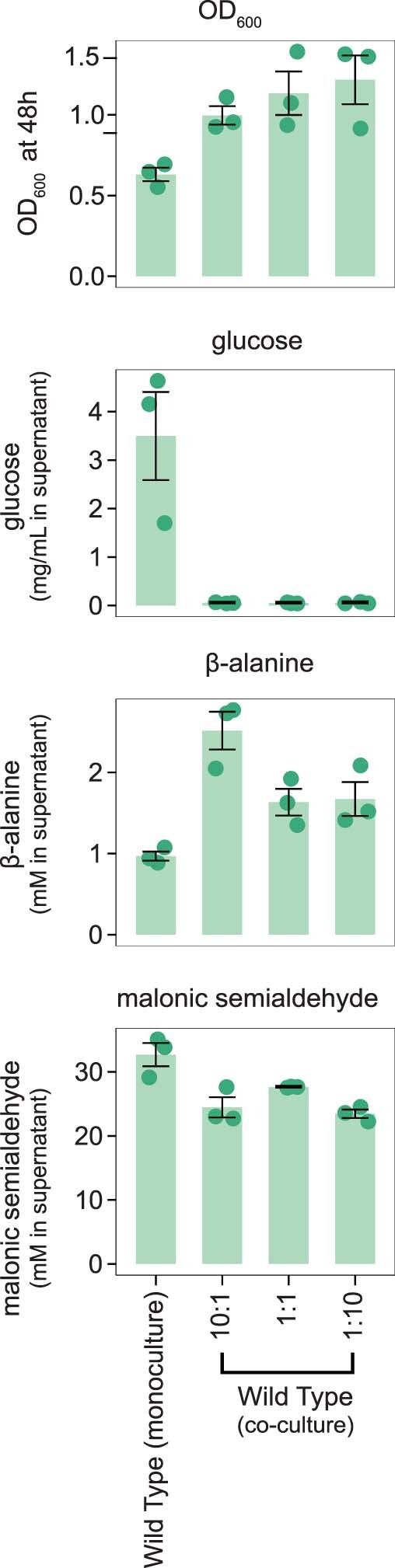
MSA production in WT monocultures and co-culture populations. Comparison of growth (OD_600)_ glucose consumption and production titres (β-alanine and MSA) of the WT monoculture (each cell expresses both enzymes) with the WT co-culture comprised of two WT strains (cells of one strain expressing *Tc*PAND and those of the other express *Bc*BAPAT) inoculated at different ratios (1:10, 1:1 and 10:1), at 24 h. Error bars denote standard error around the mean. Source data

**Extended Data Fig. 10:**
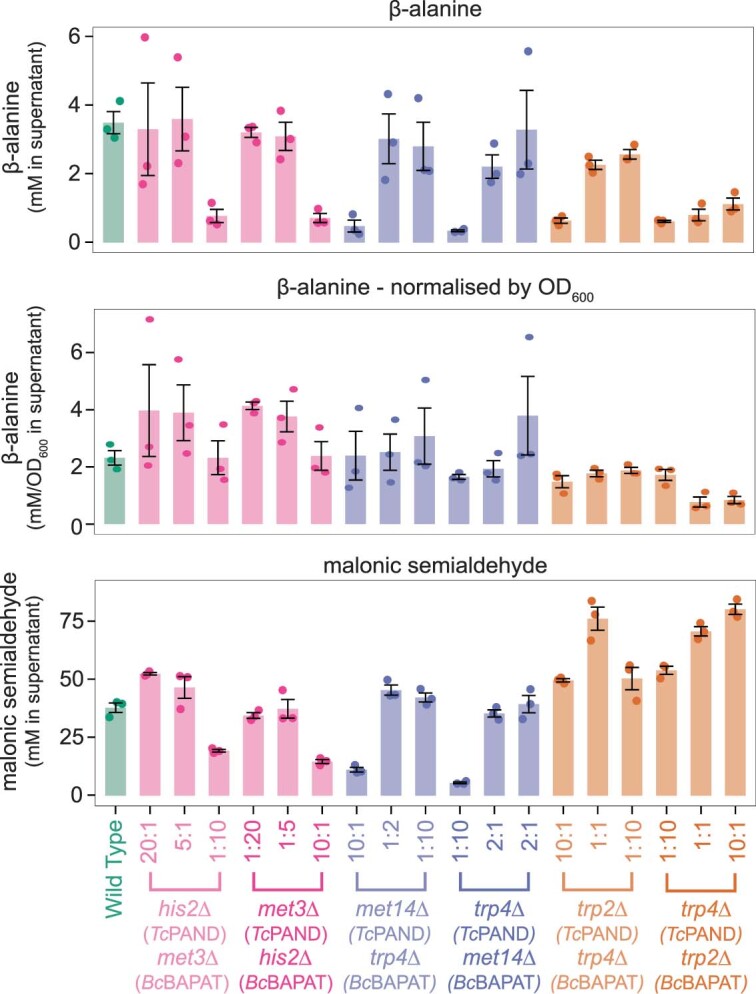
β-Alanine quantification in the WT (BY4741 + pHLUM strain) monoculture compared to all syntrophic co-cultures at 48 h. Extracellular concentrations of β-alanine (top—concentration in supernatant, middle—concentration in supernatant normalized by OD_600_) and malonic semialdehyde (MSA) in WT cells and all engineered co-cultures bearing the MSA biosynthesis pathway. Source data

Next, we cloned the genes encoding *Tc*PAND and *Bc*BAPAT into three of the validated pairs—each with distinct population ratios (one enzyme per constituent auxotroph; Fig. 5b). We also swapped the genes that were cloned into each constituent auxotroph to form the ‘reverse’ cocultures. Then, we compared OD_600_, glucose consumption, β-alanine and MSA (at 24 h and 48 h) of the WT monoculture with the syntropic cocultures bearing the split MSA biosynthesis pathway for three different inoculation ratios of the constituent auxotrophs per coculture (Fig. 5c and Extended Data Figs. 9 and 10). As expected, we found that both growth and production varied substantially with changes in the inoculation ratios. OD_600_ values changed inversely to glucose consumption levels, and the main contributor to OD_600_ values seems to be the syntrophic relationship, as final ODs are similar regardless of which gene of the pathway is being expressed in each auxotroph. Notably, one of the arrangements for the *trp2*Δ–*trp4*Δ coculture reached a twofold increase in the absolute titer compared to the monoculture. Furthermore, when we compared relative production per unit biomass, all cocultures outperformed the monoculture by a factor of up to six times (Fig. 5e and Supplementary Data 8).

Thus, we report here, to the best of our knowledge, the first demonstration of an improvement in the yield of a heterologous biosynthetic pathway using spontaneously cooperating, syntrophic, intraspecies yeast deletion mutants. In addition, for all the successful cocultures, we report a variety of population dynamics between the constituent auxotrophs (Fig. 2) ranging from an extreme predominance by one strain (such as the *trp2*Δ*–trp4*Δ coculture) to balanced populations (such as the *met14*Δ*–trp4*Δ coculture). Such a variety of population dynamics within the repertoire of available cocultures reported here is a valuable tool for biotechnological applications. Because each heterologous biosynthesis pathway could require a different optimum metabolic flux distribution in the coculture, different ratios of the auxotrophs bearing subparts of the metabolic pathway could be used to achieve optimal product yield.

## Discussion

Auxotrophy, defined as the dependence of a mutant organism on an additional and externally supplied nutrient for its growth, has a long history of use in both basic and applied yeast research^30^. The creation of auxotrophic selection markers for *S. cerevisiae* enabled decades of ground-breaking discoveries and contributed to the popularity of this species as a model organism for work on eukaryotic metabolism^30^. Yeast strains bearing complementary auxotrophies however appeared to be incapable of compensating for these defects and surviving as a community in minimal culture media, creating a paradigm that yeast cells might generally lack sufficient metabolite export to enter syntrophy. While for most of their history, outliers to this well-established paradigm were treated as problematic exceptions, recent work on intraspecies metabolic cooperation has shed fresh light on the consequences of such interactions, both for fundamental research and biotechnology^18,22,30,37^.

In the field of microbial biotechnology, such natural or engineered metabolic cooperation between auxotrophs is viewed as an increasingly attractive tool due to multiple advantageous features of microbial communities^18^. The introduction of new functionalities (often conferred by the introduction of heterologous pathways) poses many challenges such as additional metabolic demands on the host cell (for example, higher ATP or reducing equivalent requirements) and can result in altered metabolic flux distributions that can compromise the delicate balance of flux in metabolic networks and results in low product yields^43^. Because metabolically cooperative communities enable the division of labor among community members and have been demonstrated to increase robustness to environmental perturbations, they offer potential solutions to such metabolic and strain engineering problems^44,45^. Indeed, recent work by various groups has reported improvements in biosynthetic yield (ethanol^46^, butanol^47^, muconic acid^48^, flavonoids^49^, oxygenated isoprenoids^13^, oxygenated taxanes^13^, advanced biofuels^50^ among others^51^), substrate degradation (dibenzothiophene^23^, parathion^21,52,53^) and the creation of metabolic pathways unfeasible in a single organism (mini-cellulosomes^54,55^, simultaneous use of carbon sources^56,57^) by dividing pathways into modules introduced into distinct intraspecies or interspecies cell types. However, the creation of these cooperative communities has, thus far, required extensive efforts to enforce commensalistic or mutualistic interactions between different cell types within the community^11,51,58^, as the fundamental problem of the strain or species with a higher growth rate out-competing a potential metabolic partner has to be overcome for the community to establish. Thus, besides the extensive time and engineering costs involved, controlling community populations by using synthetically designed circuits has the additional caveat of being prone to genetic reversion. Therefore, the ability to establish stable syntrophy without extensive manipulation of genetic circuits would help overcome a major strain design and engineering bottleneck. Here we attempted to address this bottleneck by discovering spontaneously establishing syntrophic communities of *S. cerevisiae*.

Although the paradigm holds that common laboratory auxotrophs of *S. cerevisiae* do not spontaneously enter syntrophic growth without either (1) additional genetic manipulation^25^ or (2) by allowing the cooperation to establish progressively by plasmid lost as in SeMeCos^31^, to our knowledge, only a small number of widely used auxotrophic markers has been tested before this work. We aimed to fill this gap by undertaking a genome-scale screen that could comprehensively assemble microbial consortia and characterize cross-feeding relationships among pairs of auxotrophs. We tested 1,891 pairs of *S. cerevisiae* auxotrophs in a genome-spanning prototrophic gene-deletion collection^32^. In 97.4% of the cases, we observe no syntrophic growth. We speculate that different biological mechanisms underlie this general result. It is plausible that, in at least some cases, the metabolites to be shared are not produced or exported in enough quantity. Previous work with SeMeCo communities also indicates that other factors, such as the kinetics of metabolite exchange, could be at play. For instance, if a metabolite is depleted before syntrophy can be established, the community will inevitably collapse^31^. However, a small fraction (2.6%) of the tested auxotrophic combinations could overcome these obstacles and spontaneously formed stable, syntrophic communities, just upon mixing and without additional manipulations. In addition, we demonstrated the potential of these newly discovered communities to increase production titers of a metabolite of industrial interest. Because our primary screen only tested for spontaneous community formation of pairs of auxotrophs inoculated at an initial ratio of 1:1, putative hits that would have thrived in other ratios may have been missed. Thus, further expanding yeast coculturing screens by combining higher numbers of strains and expanding the range of initial inoculation ratios holds great potential.

Notably, some of our validated syntrophic cocultures showed extremely skewed population distributions. This is counter-intuitive, and at first glance, the results appear like that of a competition experiment between the two auxotrophs, one of which will eventually out-compete the other. However, as our experiment to assess the stability of these cocultures indicates (Fig. 3), this was not the case. Both auxotrophs retained the capability of growing and re-establishing the skewed population ratios upon re-inoculation and consequent dilution in fresh media. Indeed, one of the auxotroph pairs with the most unbalanced ratio (*trp2∆–trp4∆*) was also the one that led to the highest production titers when we engineered a heterologous pathway for MSA biosynthesis into each auxotroph pair (Fig. 5). This observation indicates that both strains in this community cooperate despite being present in very different ratios, suggesting it is metabolite export and import rates of the exchanged metabolites, rather than the maximum specific growth rates, that determine the community composition. Because this community comprises strains bearing deletions of two different genes (*TRP2* and *TRP4*) within the tryptophan biosynthesis pathway, a biosynthetic intermediate was the likely candidate for a metabolite to be exchanged between these two strains. We identified anthranilate (and possibly indole) as the intermediate being exchanged by the *trp2∆–trp4∆* community. Indeed, previous work on *S. cerevisiae* involving auxotrophs bearing deletions in the tryptophan biosynthesis pathway and studies employing various environmental cues have demonstrated the secretion and accumulation of anthranilate^59,60^. Thus, the *trp2∆–trp4∆* community reveals that syntrophic interactions can involve metabolic intermediates. This indicates that the space of potential metabolic interactions between cells is much larger than the spectrum of pathway products, such as amino acids and nucleotides. This result may be of importance for biotechnology, because for facilitating the sharing of labor between cells, it is often the intermediates and not the products that are to be exchanged.

Finally, the fact that the yield of MSA can be improved both in terms of molecules of MSA per unit biomass and the total MSA concentration in the culture, simply by co-inoculating two auxotrophs bearing one heterologous enzyme each, demonstrates the direct applicability of such coculture systems in industrial biotechnology. Indeed, we observed a trade-off between the growth rate of coculture and the production titer. Although the growth of each of the cocultures was lower than the WT, likely due to the interdependence of the two auxotrophs on each other, the total concentration of MSA produced by many of the communities was higher. Because no other strain optimization was conducted to improve the MSA yield, future genetic engineering efforts could be developed to further increase production yields from the coculture. Furthermore, because we used a simple, two-step biosynthesis pathway, splitting other pathways that require multiple, costly, heterologous enzymes between the auxotroph pairs identified here could result in higher improvements in production titers.

We hope this study will set a precedent for the use of host strain selection via high-throughput screening in the design of metabolic communities for biotechnological applications. Our work exemplifies a universal framework that can be applied to other organisms, microbial collections or conditions. In addition, the set of spontaneously establishing syntrophic yeast communities that we have discovered, and which present different features and behaviors, could serve as a valuable resource to elucidate the underlying principles of successful cross-feeding and can be directly employed to engineer microbial communities for various applications.

## Methods

### Adapting the YKO collection for growth complementation assays

The Saccharomyces Genome Deletion Project was an international effort to create a yeast deletion collection, in which ~33% of open reading frames within *S. cerevisiae* were systematically deleted using a PCR-based strategy^61^. The resulting YKO collection contains thousands of deletion strains, which have been well explored in subfields of yeast biology. Parental cell lines of the YKO are of BY4741 background—a commonly used haploid deletion strain with the following four auxotrophic alleles: *his3*Δ1, *leu2*Δ0, *met17*Δ0 and *ura3*Δ0 (ref. ^30^). Systematically deleting genes within BY4741 resulted in thousands of strains with the following five deletion mutations: *his3*Δ1, *leu2*Δ0, *met17*Δ0, *ura3*Δ0 and one other that distinguished each mutant.

Despite the YKO collection enabling unprecedented studies in functional genomics^30^, mutants with five genes deleted by design can be problematic for cross-feeding studies. When considering growth complementation assays, the rich media required for strains of the YKO collection obscure whether an auxotroph is sustained by the secretions of a neighboring strain or simply the nutrient-rich environment. At a minimum, BY4741-derived deletion strains must be supplemented with histidine, leucine, uracil and methionine when auxotrophic markers are left uncomplemented. The many auxotrophies in BY4741 make pairwise consortium testing impossible among the YKO collection, as no combination of two BY4741-derived strains can produce a collectively sufficient metabolome in commonly used SM media (YNB, 6.8 g l^−1^; glucose, 20 g l^−1^ (2%)).

We used a modified YKO collection containing the pHLUM v2 minichromosome constructed as discussed in ref. ^32^. pHLUM contains the genes *HIS3*, *LEU2*, *MET17* and *URA3*, which partially restores the genetic background of BY4741 (Extended Data Fig. 1a). Partial restoration of WT alleles (4 of 5) made BY4741-derived deletion strains suitable for pairwise consortium testing. Because BY4741-derived strains with pHLUM do not require histidine, leucine, methionine or uracil to grow in minimal media, any observed growth deficiencies can be linked to the single, remaining and uncomplemented deletion mutation. In theory, some combinations of BY4741-derived strains with pHLUM can be cocultured to produce a collectively sufficient metabolome, in which the secretion profiles of each mutant can accommodate the metabolic deficit of the other.

Deletion mutations in the YKO collection targeted nonessential genes of the YPD medium, which produced yeasts with a range of growth defects in rich and minimal media. We sought to curate a subset of BY4741-derived strains (complemented with pHLUM) with deleted loci that were essential for growth in minimal media but nonessential for growth in rich media. We referenced multiple previous works with BY4741-derived strains^31^ to assemble a preliminary library of 157 deletion strains (Extended Data Fig. 1b). Among this preliminary library, we isolated a cohort of 92 deletion strains that grew comparable to prototrophic controls (data not shown) in rich media but poorly in minimal media (Extended Data Fig. 1c). A microbial library of 92 strains could fit on a single 96-well microplate alongside four prototrophic reference strains (that is, positive controls). These 92 plus 4 BY4741-derived strains with pHLUM were selected for our pilot coculturing screen and then subjected to growth complementation assays. Coculturing BY4741-derived auxotrophs in minimal media imposes a ‘sink or swim’ scenario, in which neighboring cell populations must spontaneously cooperate by cross-feeding for collective growth. Our study sought to discover yeast strains that grew substantially better in mixed cultures than in corresponding pure cultures.

### Workflow for high-throughput growth complementation assays

A Biomek NXP (Beckman Coulter, A31841) was used for all liquid-handling operations, and customized scripts were written directly on Biomek’s proprietary software interface. Operations that required colony picking were conducted with the Singer Rotor HDA (Singer Instruments), and pin pads were maneuvered by interfacing with the Singer’s user interface. All coordinated pinning strategies were executed using this Singer software’s ‘manual mode’ (Extended Data Fig. 2 and Supplementary Table 6). Initial configuration of the ‘screen-ready’ library was conducted with the automated single colony picker Stinger (a modular extension to the Singer Rotor by Singer Instruments).

Two days before coculturing, 96-well plates were labeled and filled with 200 μl of SC-His (MP Biomedicals, 114410222) using the Biomek NXP. Because the strain library contained some histidine auxotrophs, selected wells among the plates were alternatively filled with SC-Ura (MP Biomedicals, 114410622). Using the Singer Rotor, 96 distinct strains (an array of colonies on solid agar) were transferred in parallel into 2 of the 5 input plates. The three single strains (to be crossed with the library) were each inoculated into Falcon tubes containing 20 ml of SC-His/SC-Ura media, and then they were distributed into the remaining three input plates using a sterile multichannel pipette in a fume hood (200 μl per well). The five freshly inoculated input plates were placed in a shaking incubator (1030 r.p.m. at 30 °C) for 48 h.

On the first day of coculturing, 384-well plates were labeled and filled with 50 μl of SM (6.7 g l^−1^ yeast nitrogen base (Sigma-Aldrich, Y1251) with 2% (20 g per 100 ml) glucose (Sigma-Aldrich, G8270)) media using the Biomek NXP. The now-confluent input plates were removed from the shaking incubator and spun down (400*g*, 1 min) to remove all liquid droplets attached to the Breathe-Easy sealing films (VWR, 10141-844). Using the Biomek NXP, all input plates were subjected to three serial wash steps that consisted of spinning down plates to pellet cells (400*g*, 1 min), aspirating 90% of media and dispensing 180 μl of SM media into wells (which restored the original volume of 200 μl). These wash steps resulted in a 1000× media dilution (intending to flush-out residual nutrients from SC media minus histidine (SC-His)/SC media minus uracil (SC-Ura)). SILVERseal films (Millipore Sigma, Z617601-100EA) were then applied to all plates during vortexing (cell resuspension) before the plates were placed in the Tecan Infinite M200 PRO to determine OD_600_ (10 flashes/read). Values were compared, and then plates were either diluted with SM media or concentrated appropriately (using the Biomek NXP) to normalize all wells across each plate—adjusting OD readings to 2.5. At this point, each of the three input plates was divided into two. Given the physical constraints of the Singer Rotor, such plate duplication was a necessary feature of the ‘coordinated pining strategy’ (Extended Data Fig. 2). Specifically, these plate copies ensured that all strains and cocultures reached their output plate destination without any back/cross-contamination.

The Singer Rotor was manually operated to produce five output plates from eight input plates (two input plates with a microbial library and six input plates with test strains). Overall, 4 μl (sourced from one or more input wells) were transferred into every output well (containing 50 μl of SM media). By combining this 12.5× dilution with the previously applied 1,000× dilution, freshly pinned monocultures and cocultures experienced a 12,500× media dilution throughout the physical workflow. Furthermore, all wells across all five output plates were inoculated with 0.1 OD_600_-equivalents of biomass. OD of all five output plates was measured at time point 0 on the TECAN plate reader before having Breathe-Easy sealing film applied and placed in a standing incubator (30 °C). OD of all five output plates was measured again at 48 h.

Strains were grown in YPD (2% (wt/vol) glucose (Sigma-Aldrich, G8270), 20 g l^−1^ peptone (Bacto, 211677) and 10 g l^−1^ yeast extract (Bacto, 212750)); SM media (2% (wt/vol) glucose and 6.7 g l^−1^ yeast nitrogen base without amino acids (Sigma-Aldrich, Y1251)); SC-His (2% (wt/vol) glucose, 6.8 g l^−1^ yeast nitrogen base, 0.56 g l^−1^ CSM-His-Leu-Met-Trp-Ura (powder; MP Biomedicals), 60 mg l^−1^ leucine, 20 mg l^−1^ methionine, 40 mg l^−1^ tryptophan and 20 mg l^−1^ uracil) or SC-Ura (2% (wt/vol) glucose, 6.8 g l^−1^ yeast nitrogen base, 0.56 g l^−1^ CSM-His-Leu-Met-Trp-Ura (powder; MP Biomedicals, 114550422), 60 mg l^−1^ leucine (Thermo Fisher Scientific, AC125125000), 20 mg l^−1^ methionine (Thermo Fisher Scientific, A1031836), 40 mg l^−1^ tryptophan (Thermo Fisher Scientific, 140591000) and 20 mg l^−1^ histidine (Thermo Fisher Scientific, 166155000)). Strains were kept frozen or maintained on solid agar PLUSPLATES (Singer Instruments, PLU-003) throughout the course of the screen.

### Analytical pipeline for growth complementation assays

We developed an analytical pipeline to differentiate among experimental screen data and isolate growth signatures indicative of metabolic cross-feeding. Our analysis methods could parse OD_600_ datasets, account for assay-specific spatial (regional plate) bias, conduct assay quality assessments and categorize cocultures by their growth performance (Extended Data Figs. 2–5). The pipeline relies on statistical models and conditional statements (for example, median absolute deviation, Z-factors and univariate pattern recognition) to convert plate reader files into data tables (Supplementary Note). Data tables contained annotations and quality metrics that detailed how cocultures grew compared to the associated monocultures, which were then referenced for hit selection. The analytical pipeline immediately follows the physical workflow and can process thousands of growth complementation assays in minutes. Experimental screen data included cell growth (OD_600_) at 0 and 48 h among 384-well microplates. Possible culture conditions were monocultures, cocultures or blank wells. All analysis scripts related to the pipeline were written in the R programming language. The script itself and its rationale can be found in Supplementary Note. Required packages included the following: tidyverse, grid, gridExtra, stringr, xlsx, reshape2, ggrepel, datatable, userfriendlyscience and gtools. Information regarding each package’s use can be found within the CRAN repository. All Gene Ontology Terms were referenced from the Saccharomyces Genome Database.

### Physical workflow and dataset reduction

Our pilot coculturing screen included 14 batches of the physical workflow (Fig. 1a) that were performed over a 2-week period to prepare 4,186 growth complementation assays. Each batch was designed to process three test strains, which were each cocultured across a library (*n* = 92) of putative auxotrophs (plus four prototrophic reference strains). Although 4,186 assays were seeded, only a subset (1,891) was considered during hit selection. This reduction was a feature of upstream quality control steps—removing samples and corresponding assays with either high replicate spread or indications of contamination. Our analytical pipeline marked 26% of assays as having interplate positional bias (Extended Data Fig. 4b). Assay-specific spatial bias among experimental screen data may be attributed to handling procedures during microplate processing. Sealing films were applied onto microplates before and after each OD_600_ measurement, and the removal of seals may have caused an increase in cross-well contamination.

Growth complementation assays were also not considered if strains grew well in monoculture (Extended Data Fig. 4d). High-growing monocultures were the greatest cause of dataset reduction, in which 48% of growth complementation assays were marked as having inadequate activity range (Extended Data Fig. 4e). When considering windows of separation, quality is usually governed by the curated microbial library. For example, it was discovered that 27% of our input strains were leaky auxotrophs, which caused the analytical pipeline’s Z-factor assessment to consistently remove assays containing the leaky-auxotroph strains from each round. For example, although strains with deletions of *CCS1*, *FUN12*, *PRO2*, *PHA2* and *BAS1* failed to grow in minimal media after 18 h (Extended Data Fig. 1b), these strains exhibited leaky growth at 48 h and were crossed with our microbial library in batches 4, 6, 7, 9 and 10 (which contributed to lower assay yields in those rounds). Overall, including less leaky auxotrophs in the microbial library would lead to fewer assays being labeled with poor activity ranges.

Although strict thresholding in the presented analytical pipeline omitted a considerable number of consortia from downstream hit selection (Fig. 2), it enabled the use of lower-quality libraries to detect cocultures with high confidence, making our coculturing screening method compatible with a variety of microbial collections. The pipeline employs robust statistical measures to circumvent the laborious and time-consuming manual curation of microbial libraries, to deliver a small group of candidates that are more likely to be confirmed as true positives by subsequent validation efforts.

### Growth curves to characterize hits from coculture screen

Cell growth during the coculture screen was measured using a TECAN Spark set to 30 °C with measurements set to record OD_600_, with 10 flashes/read. A kinetic interval was set so that OD_600_ measurements would be taken every 15 min over the course of 48 h.

### Construction of yeast strains

For the construction of yeast strains with single gene knockouts, *S. cerevisiae* BY4741 (*MATa*, *met15*Δ, *his3*Δ, *ura3*Δ, *leu2*Δ) was used as the parental strain. For each knockout strain, the region of the target gene to be deleted was amplified by PCR from genomic DNA extracted from the corresponding mutant of the YKO collection. The resulting PCR fragment contained a KanMX4 cassette, encoding for geneticin resistance (1506 bp) along with both flanking UP-Tag and Down-Tags (166 bp), and locus-dependent homologous ends. KanMX4 amplicons were purified and used to transform BY4741 cells. Transformed cells were then plated on YPD agar plates supplemented with 500 µg ml^−1^ geneticin (Thermo Fisher Scientific, 10131035). Individual colonies were picked, and successful deletion of the target gene was confirmed by performing colony PCR (Phire Plant Direct reaction mix; Thermo Fisher Scientific, F160L) with primers targeting the corresponding flanking regions, as well as primers designed to bind to the *kanMX4* resistance cassette (see Supplementary Table 7 for list of primers).

For the construction of auxotrophic yeast strains with either mTAGBFP2 or mScarlet-I, first, the genes encoding both fluorescent proteins were cloned into the pWS064 vector, which carries a copy of the *LEU2* gene next to the insertion site of the gene of interest. The appropriate auxotrophic yeast strains were then transformed with the vector carrying the desired fluorescence and pHUM and plated on plates of SC medium lacking uracil and leucine to select transformants of pHUM where the fluorescence gene had integrated successfully. Colonies were picked and replated onto SC plates lacking uracil and leucine, repeating the process three times. Final colonies were picked, and the integration of fluorescence genes was verified by colony PCR with primers targeting internal regions of the exogenous genes. Details of all plasmids, synthetic DNA and strains used in the study can be found in Supplementary Tables 7–10.

For the construction of yeast strains for the production of MSA, first, plasmids carrying the necessary coding sequences, promoters and terminators were assembled using the Yeast ToolKit modular assembly system, using previously published protocols^62^.

Briefly, all the synthetic individual genes with the appropriate overhangs were cloned into level 0 vector pYTK001. Golden Gate was used to clone the gene encoding *Tc*PAND (*LOC100124592*) under the promoter pTDH3 and terminator tADH1 into vector pYTK096, which carries a copy of the *URA3* gene. A similar strategy was used to clone the gene encoding *Bc*BAPAT, with promoter pTDH3 and terminator tADH1 into the vector pWS041. Both level 1 plasmids were then assembled into vector pYTK096 for the simultaneous expression of both genes. Finally, the full pathway comprising the corresponding level 0 plasmids of each gene was assembled into level 1 vectors pWS041 and pWS043, respectively, in both cases with promoter pTDH3 and terminator tADH1. Then, all level 1 plasmids were assembled into vector pYTK096. The cassettes expressing the genes encoding *Tc*PAND, BcBAPAT or both were used to transform the target yeast strain derived from BY4741. Cells were also transformed with pHLM. Transformed cells were plated onto SC plates lacking uracil and leucine to select transformants of pHLM where the genes for MSA production had integrated successfully. Colonies were picked and replated onto SC plates lacking uracil and leucine, repeating the process three times. Final colonies were picked and integration of the genes for the production of MSA was verified by colony PCR with primers targeting internal regions of the exogenous genes.

### Fluorescence analysis of yeast cocultures

Auxotrophic strains tagged with mTAGBFP2 and mScarlet-I were transformed with pHUM and grown overnight in SC medium lacking uracil and leucine. Overnight cultures were washed three times by spinning culture tubes to pellet cells at 2,500*g* for 10 min, removing the supernatant, and resuspending in 1× PBS (3 ml). Cells were resuspended in yeast nitrogen base after the final wash. Nine serial dilutions of each monoculture, representing optical densities of 0.95, 0.90, 0.80, 0.66, 0.5, 0.33, 0.20, 0.10 and 0.05, were prepared. Cocultures were then inoculated in the range of ratios (1:20, 1:10, 1:5, 1:2, 1:1, 2:1, 5:1, 10:1 and 20:1), and the final combined OD_600_ of the inoculum of each coculture was 0.10. In total, 100 μl of each coculture was transferred to 96-well plates in triplicates. At each time point, pure monocultures of the auxotrophic fluorescent strains in SC medium were pelleted and resuspended in yeast nitrogen base at several different OD_600_, and the fluorescent intensity of each dilution was measured to generate calibration curves for each fluorescent strain. Population dynamics of cocultures were tracked with a Spark Tecan (600 nm range, 20 flashes/read and kinetic interval: 20 min), where absorbance, mTAGBFP2 (excitation 400 nm and emission 465 nm), and mScarlet-I (excitation 560 nm and emission 620 nm) were monitored in parallel.

Cocultures were also analyzed by fluorescence microscopy. Samples from each culture, cultivated as described above, were extracted at 0, 24, 48 and 72 h (in the case of monocultures, only at 0 h) and fixed by adding paraformaldehyde at a final concentration of 4 g l^−1^ in 3.6% sucrose. After 15 min, fixed cells were centrifuged at 4,000*g* for 10 min and washed four times in 1× PBS at an OD_600_ of 1. Then, four different volumes (5, 10, 15 and 20 μl) of each fixed sample were transferred to poly-lysine coated 384-well glass-bottomed imaging plates (CellCarrier Ultra; PerkinElmer, 6055300), and the plates were imaged on a PerkinElmer Opera Phenix High Content Screening System (with ×40 water immersion objective, a numerical aperture of 1.1, in confocal mode). Single plane images in brightfield, blue fluorescence (excitation 405 nm and emission 435–480 nm) and red fluorescence (excitation 561 nm and emission 650–760 nm) channels were acquired for 29 fields for each well. Images were analyzed and percentages of blue and red cells were calculated with PerkinElmer Harmony software (version 4.9).

### LC–MS-based quantification of anthranilate and tryptophan

All strains were precultured in SC media (Sigma-Aldrich, Y2001; with added histidine (20 mg l^−1^), leucine (60 mg l^−1^), tryptophan (40 mg l^−1^) and uracil (20 mg l^−1^) and 2% glucose) for 14 h and washed three times with Millipore H_2_O. After washing, each strain was inoculated into the final culture media (SM—yeast nitrogen base (Sigma-Aldrich, Y0626) with 2% glucose), SM + tryptophan (40 mg l^−1^) or SM + anthranilate (Thermo Fisher Scientific, A15681.30) such that the initial OD of each culture was 0.2. After 8 h of growth, the cultures were centrifuged (1,200*g*, 25 °C, 5 min). The supernatant was filtered through a 0.2 µm syringe filter and lyophilized. The lyophilized samples were reconstituted in 0.5 ml of Millipore water to achieve a 20× concentrated solution of the original supernatant. Ten microliters of this solution were used for derivatization using benzoyl chloride^63^. In total, 10 µl of 100 mM aqueous Na_2_CO_3_ (Sigma-Aldrich, 223530) and 20 µl of 2% benzoyl chloride (Sigma-Aldrich, 259950) in acetonitrile (Sigma-Aldrich, 34851; freshly prepared) were added sequentially to a 500 µl vial containing 10 µl of the reconstituted lyophilized supernatant solution. Following a brief mixing (5 s) and incubation (1 min), the samples were centrifuged (14,800*g*, 25 °C, 10 min) and 30 µl was transferred to LC–MS amber vials with glass insert and stored at 4 °C for LC–MS analysis. An external calibration standard containing commercially available tryptophan, indole and anthranilate, each in 1 mM concentration, was prepared in millipore water and derivatized as above. The derivatized calibration standards were subsequently diluted in the ratio 1:4:4:4:4:4:4:4 using 50% acetonitrile in water. To measure recovery, samples were prepared by combining a suitable control yeast culture supernatant sample (10 µl) with 2 µl of the calibration standard mix followed by derivatization as above.

LC–MS measurement was carried out on Agilent Infinity 1290 high-performance liquid chromatography (HPLC) coupled to Agilent 6460 triple quadrupole mass spectrometer. The LC parameters are as follows: solvents A and B were 10 mM aqueous ammonium formate containing 0.1% formic acid and 100% acetonitrile, respectively. The chromatography was carried out using an Agilent Eclipse Plus C18 column (3.0 × 50 mm) maintained at 30 °C and a flow rate of 0.3 ml min^−1^. The applied solvent composition consisted of 50% B from 0 to 3.9 min followed by 100% B from 4 min to 6 min. The column was then re-equilibrated at 50% B from 6.1 min to 7.5 min. The MS parameters are as follows: gas flow at 8 l min^−1^ and 30 °C, sheath gas flow at 11 l min^−1^ and 30 °C, nebulizer pressure at 50 psi, capillary voltage at 3,000 V (negative) and 3,500 V (positive) and nozzle voltage at 500 V. Cell acceleration voltage was set at 7 V. The analysis was carried out as dynamic multiple-reaction monitoring in the positive mode for the transitions listed in Supplementary Data 6. The raw data files from the mass spectrometer were processed using Quantitative Analysis for QQQ software.

### LC–MS quantification of β-alanine and MSA

Overnight inoculated monocultures were washed three times, OD values were diluted to 10 for each strain and then cocultures were prepared at an initial OD_600_ of 0.1 with different ratios using a 2,000 µl system (1,980 µl SM + 20 µl OD10 cells) in a 48-well deep plate. Cocultures were kept at 30 °C, 250 r.p.m., and 200 µl samples were taken at 24 h, 48 h and 72 h to check the concentrations of glucose, β-alanine and MSA, respectively.

Glucose concentration was analyzed by HPLC—100 µl cell culture was mixed with pure water to dilute two times, centrifuged at 2,000*g* for 10 min and then 200 µl supernatants were ready for HPLC analysis. The HPLC (Agilent LC1260 infinity) was equipped with a refractive index detector (Agilent Technologies) and a PL Hi-Plex H column (Varian) at 65 °C. The mobile phase was 5 mM H_2_SO_4_ at a flow rate of 0.6 ml min^−1^ (ref. ^64^).

For β-alanine and MSA analysis, 100 µl cell culture was mixed with 400 µl (50%) acetonitrile and centrifuged at 2,000*g* for 30 min, then 200 µl supernatants were transferred to a 96-well plate for LC–MS analysis and the samples were diluted five times. An Agilent 1290 Infinity system was employed to analyze these prepared samples in combination with an Agilent 6550 quadrupole time-of-flight mass spectrometer. An Agilent Poroshell 120 HILIC-Z, 2.1 × 100 mm, 1.9 µm, column was used at a temperature of 45 °C with a solvent flow rate of 0.25 ml min^−1^. LC separation was performed with buffer A (10 mM ammonium formate in water) and buffer B (10 mM ammonium formate in water:ACN 10:90 (vol:vol)). After 0.5 min at 98% B, the composition was changed to 5% buffer B over 2.5 min, then held at 5% buffer B for 1 min. Injection volume was 1 μl, and negative ion spectra were recorded over a mass range of 100–1000 *m/z* at a rate of 1 spectrum per second. β-Alanine was quantified by the prepared calibration curve of the β-alanine standard, while MSA was semi-quantified by the functional *m/z* values and the standard curves of β-alanine only due to the shortage of extremely expensive MSA standard. The results were analyzed with Agilent MassHunter Qualitative Analysis.

### Reporting summary

Further information on research design is available in the Nature Portfolio Reporting Summary linked to this article.

## Online content

Any methods, additional references, Nature Portfolio reporting summaries, source data, extended data, supplementary information, acknowledgements, peer review information; details of author contributions and competing interests; and statements of data and code availability are available at 10.1038/s41589-023-01341-2.

## Supplementary information

Supplementary Information Supplementary Note and Tables 1–10.

Reporting Summary

Supplementary Data 1 All growth screen data (monocultures and cocultures) that passed QC thresholds.

Supplementary Data 2 Fold change of cocultures compared to fittest monocultures before *Z*-factor filtering during the primary growth complementation screen.

Supplementary Data 3 All *Z* factors from the growth screen.

Supplementary Data 4 Results of statistical analysis to evaluate syntrophy (*P* values and FC of coculture growth versus monoculture).

Supplementary Data 5 Stability of cocultures (OD_600_ changes upon subculturing for passages 1 and 2).

Supplementary Data 6 Results of LC–MS quantification of anthranilate, tryptophan and indole.

Supplementary Data 7 Results of LC–MS-based quantification of MSA bioproduction.

Supplementary Data 8 Results of LC–MS-based quantification of MSA bioproduction—normalized by OD_600_.

## Data Availability

All data generated or analyzed during this study are included in this published article and its supplementary data or source data files. Source data are provided with this paper.

## Source data

Source Data Fig. 1 Statistical source data.
Source Data Fig. 2 Fluorescence and OD data.
Source Data Fig. 3 Fluorescence and OD data.
Source Data Fig. 4 OD and metabolite quantification data.
Source Data Fig. 5 OD and metabolite quantification data.
Source Data Extended Data Fig. 1 OD data for auxotrophs.
Source Data Extended Data Fig. 5 Statistical source data.
Source Data Extended Data Fig. 6 Fluorescence and OD data.
Source Data Extended Data Fig. 7 Fluorescence and OD data.
Source Data Extended Data Fig. 8 Metabolite quantification data.
Source Data Extended Data Fig. 9 OD and metabolite quantification data.
Source Data Extended Data Fig. 10 OD and metabolite quantification data.

## Code availability

Required packages included: tidyverse, grid, gridExtra, stringr, xlsx, reshape2, ggrepel, datatable, userfriendlyscience and gtools. Information regarding each package’s use can be found within the CRAN repository. All Gene Ontology (GO) terms were referenced from the Saccharomyces Genome Database (SGD). All code used to both process data and generate plots is available at https://github.com/Ralser-lab/auxcocul.
