# Supplementary material for: Spontaneously established syntrophic yeast communities improve bioproduction
Source: Nat Chem Biol. 2023 May 29;19(8):951–61. doi: 10.1038/s41589-023-01341-2 (PMC10374442; doi:10.1038/s41589-023-01341-2)
Supplement: Supplementary file 1 — Supplementary Note and Tables 1–10. [file 41589_2023_1341_MOESM1_ESM.pdf]

nature chemical biology

Article

https://doi.org/10.1038/s41589-023-01341-2

Spontaneously established syntrophic yeast communities improve bioproduction

Supplementary Note 1: Screen Design and Analysis

Co-culture screen overview

We developed an analytical pipeline to differentiate among experimental screen data and isolate growth signatures indicative of metabolic cross-feeding. Our analysis methods could parse OD_{600} datasets, account for regional plate bias, conduct assay quality assessments, and categorise co-cultures by their growth performance (**Extended Data Figures 3 and 4**). The pipeline relies on statistical models (e.g., median absolute deviation, Z-Factoring, and univariate pattern recognition) and conditional statements to convert plate reader files into data tables. Data tables contained annotations and quality metrics that detailed how co-cultures grew compared to associated monocultures, which were then referenced for hit selection. The analytical pipeline immediately follows the physical workflow and can process thousands of growth complementation assays in minutes. Experimental screen data included cell growth (OD_{600}) at 0 and 48 hours among 384-well microplates. Possible culture conditions were monocultures, co-cultures, or blank wells. R Packages: *tidyverse*, *grid*, *stringr*, *userfriendlyscience.*

User-Defined Functions

1. *unique_read_tecan_xls()* - translates OD_{600} data from excel sheets to Dataframes
2. *db_characterize()* - classification algorithm for replicate distributions; see Step. 3
3. *zfactor()* - takes two numeric DataFrame vectors and calculates a Z-Factor; see Step. 4

Step 1. Importing experimental screen data

Specify paths for two types of input files:

1. Plate Reader Files (spreadsheets containing OD_{600} data). Files were originally .xlsx but were imported into R Studio as .csv. Files were allocated across 3 directories:
    a. Co-culture microtiter plates (c1, c2, and c3)
    b. Monoculture microtiter plate (n1)
    c. Monoculture microtiter plate (n2)
2. Plate Maps (positional IDs). One map for each of the 3 above directories (see **Extended Data Figure 3**). These maps were reference files that matched culture conditions with plate coordinates (i.e. assigned labels to OD_{600} values).

Step 2. Accounting for errors in physical handling

Cell cultures were inoculated among wells at 0.20 OD_{600}, thus any significant deviation from 0.20 OD_{600} at the onset (e.g., greater than 1.00) suggested plate-inoculating errors or well defects. Sample growth at *Time: 0* was aggregated and analysed as a population distribution, and wells that presented extreme OD_{600} (values beyond Q3 + 1.5*IQR) were considered outliers and removed from analysis (see **Extended Data Figure 3**).

Step 3. Mitigating assay-specific plate bias and inconsistent growth

Samples within experimental screen data had at least 4 replicates, which were either pinned in one output plate (e.g. co-cultures) or across two different plates (e.g. monocultures) (**Extended Data Figure 2**). Allocating replicates across multiple plates was a consequence of pinning microbial arrays with 96-well plating pads. Although operating with 8×12 arrays enabled rapid microplate preparation, the pinning procedure introduced the risk of inter-plate positional effects (i.e., sample replicates growing differently between plates). Beyond a certain threshold, sample variation between plates is unacceptable, as it becomes impossible to know which plate is showcasing the condition's "real" growth. This spatial, systematic bias can result in data skew, which can increase the risk of statistical errors during hit selection. To mitigate the impact of plate positional effects in our experimental screen data, we applied median absolute deviation (MAD) and a distance-based algorithm to categorise ΔOD_{600} replicate distributions (see **Extended Data Figures 3** and **4**). This quality control step was used to remove culture conditions exhibiting inconsistent growth.

MAD is a robust measure of data variability in a univariate sample and was calculated for each monoculture and co-culture. MAD values were aggregated and analysed as a population distribution, and outliers (values beyond Q3 + 1.5×IQR) were sent to a distance-based classification algorithm (*db_characterize*) for pattern recognition (**Extended Data Figure 4a**). *db_characterize* helps locate assay-specific spatial bias by classifying replicate distributions, in which distances (defined as the difference in OD_{600}) were measured between all replicate points. A threshold of 1.5 units was applied to categorise every replicate-connection as being either "close" (distance under 1.5) or "far" (distance higher than 1.5). Culture conditions received a mix of "close" and "far" labels, which were then compiled into an "inlier: outlier" ratio (**Extended Data Figure 4b**). These ratios translated into specific univariate patterns, and thus enabled automatic pattern recognition of replicate distributions.

Growth complementation assays with all conditions displaying "tight" or "one anomaly" patterns were kept in the dataset for downstream analysis. In contrast, conditions with a "pairs" or "undefined distribution" pattern were discarded. The "pairs" pattern was always attributed to monocultures that grew differently between two plates—likely due to plate positional biases rather than bimodal growth behaviour.

Step 4. Assay design, quality assessments, and isolating auxotrophs

In a typical activation-based screen, unknown samples are assayed alongside positive and negative controls. These controls define the upper and lower bounds of assay activity, and samples then signal within this fixed activity range (i.e. within the window of separation between bounds). Activity ranges within the presented co-culture screen were defined by both prototrophs (fixed positive controls) and test strains in monoculture (assay-specific negative controls) (**Extended Data Figure 4d**). Prototrophic strains grew unabated in minimal media (100% assay activity), while test strains were expected to have stunted growth (0% assay activity).

It is worth noting that complementary growth is relative to the strains involved, and 0% assay activity is not identical to null OD_{600}. Strains in monoculture could grow to negligible or moderate amounts, yet their average growth was still defined as baseline activity. Monoculture growth was equated to background noise so that any additional growth among co-cultures would translate as assay activity between 0-100%. Although competitive behaviour between strains could cause co-cultures to grow less than monocultures, these consortia are not indicative of cross-feeding and thus were ignored. The described assay identifies syntrophic relationships among co-cultures when 1) prototrophic references grow well and 2) all other monocultures grow poorly (**Extended Data Figure 4e**).

Incubating hundreds of distinct consortia in parallel can produce a wide range of growth activity. Unlike traditional high-throughput screening libraries (e.g., collections of small-molecules), microbial libraries can evolve overtime and thus require active management. Some of our library strains grew unexpectedly (i.e. monocultures grew in minimal media; i.e., leaky auxotrophs) despite pre-screen efforts to curate only auxotrophs. High-growing monocultures are flagged in the presented analytical pipeline, as they cause growth complementation assays to have inadequate windows of separation between negative and positive controls (**Extended Data Figure 4e**).

We isolated auxotrophs in experimental screen data by conducting assay quality assessments. The Z-Factor is a screening window coefficient to measure effect size—a dimensionless statistic that is commonly used to evaluate assay quality in high-throughput screening (**Extended Data Figures 3** and **4e**) [8]. Windows of separation were calculated for each assay, in which variance and dynamic range were measured among test monocultures and prototrophs (producing a value between 0 and 1). Test strains that earned a Z-Factor of 0.50-1.00 were considered excellent quality, while earning less than 0.50 was interpreted as inadequate (i.e. too much signal overlap between negative and positive controls). Since test strains were considered negative controls, assay quality (i.e. separation between lower and upper bounds) was directly impacted by monoculture growth. As a consequence, isolating strains with a Z-Factor above 0.50 could select for both high-quality assays and low-growing monocultures (**Extended Data Figures 4d-e**). Therefore, high-growing monocultures are flagged and subsequently removed from downstream consideration, as they cause growth complementation assays to have inadequate windows of separation between negative and positive controls (**Extended Data Figure 4e**).

Step 5. Creating comparison groups (adding features to DataFrames)

Up to this point, samples were viewed as stand-alone observations in the dataset, with no data structure for connecting co-cultures (e.g. XY) to corresponding monocultures (e.g. X and Y). Culture conditions were assigned ‘Assay ID’ labels to bundle and filter raw data based on growth complementation assays. Experimental screen data was divided into smaller data frames (i.e., *comparison.data*) based on the test strain that each assay contained (**Extended Data Figure 3**). Each comparison DataFrame contained one of 92

distinct assays (e.g. X, Y, and XY), each of which was evaluated separately in downstream steps.

Step 6. Removing unsuitable growth complementation assays

Assays that failed to meet set criteria were removed from analysis. Steps 2-4 produced lists of flagged samples (i.e. co-cultures or monocultures with unacceptable variance, plate positional bias, median growth, or Z-Factors). These “flagged-for-removal” tables were referenced in a sequence of filtering steps to remove unsuitable assays from *comparison.data* (**Extended Data Figure 3**). Exclusion criteria included:

1. Assays with culture conditions found in flagged.t0s (i.e. outlier OD_{600} at *Time: 0*).
2. Assays with culture conditions labelled as “pairs” or “undefined” from *db.characterize.*
3. Assays with an “infeasible” activity range (i.e. monoculture earning a Z-Factor < 0.50).
4. Assays with inadequate sample size (i.e. culture conditions with less than 3 replicates)

Step 7. Characterising complementary effects in each consortium

Screening algorithms with inadequate sorting criteria will struggle to differentiate biological significance from false positives. In our specific case, for the presented co-culture screen, not all instances of complementary growth in consortia are representative of metabolic cross-feeding. Synergy (i.e. AB > A + B) is associated but not sufficient for syntrophy. Categorising assays only by the presence/absence of synergy fails to distinguish between 1) cross-feeding behaviour, or 2) one strain augmenting the growth of a self-sufficient strain (**Extended Data Figure 3**). Therefore, the novelty of a consortium depends on how constituent strains grew in monoculture. The presented analytical pipeline differentiates among experimental screen data by adding descriptive annotations to each assay, which helps contextualise, compare, and rank complementary behaviour between co-cultures and monocultures (**Extended Data Figure 3**).

Assays were broken down into their primary components: monoculture X, monoculture Y, and co-culture XY. Unequal variance t-tests determined whether the difference in growth between co-cultures and the corresponding monocultures was significant (**Extended Data Figures 3**). Tests considered ΔOD_{600} sample distributions and produced p-values in relation to the following null and alternative hypotheses:

$$H_0: \mu_{co\text{-}culture} = \mu_{monoculture}$$

***H*₁:** *Co-culture grew significantly different from its monoculture*

Two p-values were generated per assay, each of which compared a co-culture with one of its monocultures. Since larger p-values from Welch’s t-tests correspond to less confidence in samples having different means, larger p-values indirectly select for monocultures whose growth behavior was most similar to its co-culture. The median of this more ‘likened’ monoculture thus served as a growth benchmark for evaluating consortia performance (**Extended Data Figure 3**). Fold difference ratios (co-culture over monoculture) were calculated to define the magnitude of difference (i.e., statistical effect

size) between sample medians. Consortia were further annotated by their presence/absence of synergy, and the difference in growth between co-cultures and more ‘likened’ monocultures (**Extended Data Figure 3**)

Step 8. Defining a “Hit Threshold”

Growth complementation assays were mapped onto a volcano plot to visualize the log_{10}(p-value) and log_2(Fold Difference) of each co-culture (p-values were corrected for multiple testing using the Benjamini-Hochberg method.). Consortia that differentiated the most from its monocultures populated the top-right corner of the plot, and assays failing to earn p-values < 0.05 or fold difference ratios > 1.50 were not further considered (**Figure 1b**). Remaining consortia could be filtered by their metadata annotations, such as their presence of synergy and differences in OD_{600} between co-cultures and “more-likened” monocultures.

Supplementary Table 1. 62 strains from the primary screen that grow well in SC but not in SM. Strains were manually annotated based on any explicit linkages to amino acid or nucleotide biosynthesis (e.g., direct).

Condition	Associated product	Gene ontology	AA_or_Nucl_biosynth_manual_anno	Direct_or_not	Hit	Hit_and_Direct
ADE5,7	adenine	purine nucleobase biosynthesis	direct	1	1	1
ARG1	arginine	arginine biosynthesis	direct	1	1	1
ARG2	arginine	arginine biosynthesis	direct	1	1	1
ECM29	proteasome	proteasome assembly	none		1	
ERG4	ergosterol	ergosterol biosynthesis	none		0	
HIS1	histidine	histidine biosynthesis	direct	1	1	1
HIS4	histidine	histidine biosynthesis	direct	1	1	1
HIS6	histidine	histidine biosynthesis	direct	1	1	1
HMF1	unknown	unknown	unknown		0	
IBA57	protein maturation	iron-sulpher cluster	indirect		1	
ISA1	protein maturation	iron-sulpher cluster	indirect		0	
LYS1	lysine	lysine biosynthesis	direct	1	0	
MET10	methionine/sulphate	sulphate assimilation	direct	1	1	1
MET13	methionine/sulphate	methionine biosynthesis	direct	1	1	1
MET7	methionine/sulphate	methionine biosynthesis	direct	1	0	
MHF2	DNA damage response	DNA damage response	none		1	
YBR209W	unknown	unknown	unknown		0	
ORT1	arginine	ornithine transporter	direct	1	1	1
POS5	stress response	oxidative stress response	none		0	
RPL27A	ribosome	cytoplasmic translation	none		1	
TRP2	tryptophan	tryptophan biosynthesis	direct	1	1	1
TRP3	tryptophan	tryptophan biosynthesis	direct	1	0	
URA1	uracil	pyrimidine nucleobase biosynthesis	direct	1	1	1
VPS65	unknown	unknown	unknown		0	
ADE1	adenine	purine nucleobase biosynthesis	direct	1	1	1
ARG3	arginine	citruline biosynthesis	direct	1	1	1
ARO1	aromatic amino acids	chorismate biosynthesis	direct	1	1	1
ARO7	aromatic amino acids	tyrosine biosynthesis	direct	1	1	1
ILV1	isoleucine/valine	isoleucine biosynthesis	direct	1	1	1

IRC10	unknown	unknown	unknown		1	
LAG1	ceramide	ceramide biosynthesis	none		0	
LYS4	lysine	lysine biosynthesis	direct	1	1	1
LYS9	lysine	lysine biosynthesis	direct	1	0	
MET3	methionine/sulphate	sulphate assimilation	direct	1	1	1
ADE2	adenine	purine nucleobase biosynthesis	direct	1	0	
ADE6	adenine	purine nucleobase biosynthesis	direct	1	1	1
LYS2	lysine	lysine biosynthesis	direct	1	0	
MET14	methionine/sulphate	sulphate assimilation	direct	1	1	1
RPS24B	ribosome	ribosome structure	none		1	
TRP5	tryptophan	tryptophan biosynthesis	direct	1	0	
ARG4	arginine	arginine biosynthesis	direct	1	0	
ARG5,6	arginine	arginine biosynthesis	direct	1	0	
ARG8	arginine	arginine biosynthesis	direct	1	1	1
BUD16	vitamin B6	bud site selection	none		0	
FCY22	cytosine	nucleobase transport	direct	1	0	
PEX35	unknown	peroxisome associated	none		0	
RSC2	DNA translocase activity	DNA translocase activity	none		1	
TRP4	tryptophan	tryptophan biosynthesis	direct	1	1	1
HIS2	histidine	histidine biosynthesis	direct	1	1	1
LYS5	lysine	lysine biosynthesis	direct	1	0	
MET1	methionine/siroheme	siroheme biosynthesis	direct	1	1	1
YHL005C	unknown	unknown	unknown		0	
URA4	uracil	pyrimidine nucleobase biosynthesis	direct	1	0	
ADE8	adenine	purine nucleobase biosynthesis	direct	1	0	
ARO2	aromatic amino acids	chorismate biosynthesis	direct	1	1	1
LYS12	lysine	lysine biosynthesis	direct	1	1	1
YEA4	transmembrane transport	transmembrane transport	indirect		1	
MET5	methionine/sulphate	sulphate assimilation	direct	1	1	1
APT1	adenine	purine nucleobase biosynthesis	direct	1	0	
APL2	clathrin	golgi transport	indirect		0	

PRP18	mRNA splicing	mRNA splicing	none		0	
VPH2	V-ATPase assembly	vacuolar acidification	none		1	
			**Sum**	**41**		**27**

Supplementary Table 2. 49 co-cultures that showed syntrophic growth based on statistical tests from the primary screen (see Supplementary Note 1). Strains were manually annotated based on any explicit linkages to amino acid or nucleotide biosynthesis (e.g., direct).

Coculture	AA_or_Nucl_biosynth_manual_anno	Number_direct	Number_direct_or_indirect
met10Δ-his2Δ	direct	2	2
ade1Δ-yea4Δ	direct	1	2
ade1Δ-met13Δ	direct	2	2
ura1Δ-met13Δ	direct	2	2
trp4Δ-trp2Δ	direct	2	2
ade1Δ-aro2Δ	direct	2	2
lys12Δ-aro2Δ	direct	2	2
rpl27aΔ-aro2Δ	direct	1	1
ura1Δ-aro2Δ	direct	2	2
ade1Δ-arg2Δ	direct	2	2
irc10Δ-arg2Δ	direct	1	1
met1Δ-arg2Δ	direct	2	2
met14Δ-arg2Δ	direct	2	2
met3Δ-arg2Δ	direct	2	2
met5Δ-arg2Δ	direct	2	2
ade1Δ-his1Δ	direct	2	2
met5Δ-his1Δ	direct	2	2
rpl27aΔ-his1Δ	direct	1	1
ade1Δ-iba57Δ	direct	1	2
rpl27aΔ-iba57Δ	indirect	0	1
arg1Δ-rps24bΔ	direct	1	1
ecm29Δ-rps24bΔ	none	0	0
lys12Δ-rps24bΔ	direct	1	1
met13Δ-rps24bΔ	direct	1	1
mhf2Δ-rps24bΔ	none	0	0
ade1Δ-ilv1Δ	direct	2	2
arg2Δ-met14Δ	direct	2	2
arg3Δ-met14Δ	direct	2	2
aro7Δ-met14Δ	direct	2	2
his2Δ-met14Δ	direct	2	2
irc10Δ-met14Δ	direct	1	1
lys4Δ-met14Δ	direct	2	2
his4Δ-arg8Δ	direct	2	2
ade1Δ-vph2Δ	direct	1	1
ade5,7Δ-vph2Δ	direct	1	1

trp4Δ-ade6Δ	direct	2	2
his2Δ-met3Δ	direct	2	2
trp4Δ-met3Δ	direct	2	2
his2Δ-met1Δ	direct	2	2
trp4Δ-met1Δ	direct	2	2
aro2Δ-his6Δ	direct	2	2
lys12Δ-his6Δ	direct	2	2
mhf2Δ-his6Δ	direct	1	1
aro1Δ-trp4Δ	direct	2	2
his4Δ-trp4Δ	direct	2	2
irc10Δ-trp4Δ	direct	1	1
met14Δ-trp4Δ	direct	2	2
ort1Δ-trp4Δ	direct	2	2
rsc2Δ-trp4Δ	direct	1	1

Supplementary Table 3. Growth complementation assays, with OD600 and respective standard deviation, of nine recreated auxotrophic pairs.

Culture	Strain Origin	OD600_mean	OD600_std
BY4741	This study	0.0505	0.00129099
BY4741 pHLUM	This study	0.86025	0.03409179
*met3*Δ	YKO Collection	0.16685875	0.05206742
*met3*Δ	This study	0.21740083	0.01226765
*his2*Δ	YKO Collection	0.20179208	0.02665219
*his2*Δ	This study	0.22251667	0.03123918
*met3*Δ-*his2*Δ	YKO Collection	0.49151375	0.03683669
*met3*Δ-*his2*Δ	This study	0.44523417	0.02467813
*met3*Δ	YKO Collection	0.25447083	0.02838738
*met3*Δ	This study	0.20832167	0.00991154
*met1*Δ	YKO Collection	0.21594833	0.03210962
*met1*Δ	This study	0.19568667	0.00398338
*met3*Δ-*met1*Δ	YKO Collection	0.50963333	0.03596337
*met3*Δ-*met1*Δ	This study	0.54544	0.02165078
*met14*Δ	YKO Collection	0.21541792	0.00831098
*met14*Δ	This study	0.1947225	0.03887196
*met5*Δ	YKO Collection	0.21889167	0.02576922
*met5*Δ	This study	0.23153333	0.00215012
*met14*Δ-*met5*Δ	YKO Collection	0.49532708	0.06268514
*met14*Δ-*met5*Δ	This study	0.52626083	0.02021706
*met14*Δ	YKO Collection	0.22673208	0.02635929
*met14*Δ	This study	0.22167417	0.01165391
*trp4*Δ	YKO Collection	0.13165375	0.00541241
*trp4*Δ	This study	0.14359083	0.00870019
*met14*Δ-*trp4*Δ	YKO Collection	0.60557542	0.01925722
*met14*Δ-*trp4*Δ	This study	0.60988417	0.00211399
*met1*Δ	YKO Collection	0.17324167	0.03717292
*met1*Δ	This study	0.20360753	0.0115404
*arg2*Δ	YKO Collection	0.18278958	0.00122266
*arg2*Δ	This study	0.19117917	0.01910499
*met1*Δ-*arg2*Δ	YKO Collection	0.34528333	0.07468758
*met1*Δ-*arg2*Δ	This study	0.36427	0.03047448
*lys12*Δ	YKO Collection	0.2487425	0.00592643
*lys12*Δ	This study	0.22266	0.05738726
*trp4*Δ	YKO Collection	0.16916583	0.02769275
*trp4*Δ	This study	0.22668167	0.023311
*lys12*Δ-*trp4*Δ	YKO Collection	0.35610583	0.00162566
*lys12*Δ-*trp4*Δ	This study	0.40814667	0.01534124

met3Δ	YKO Collection	0.1805	0.03049044
met3Δ	This study	0.21025	0.0447167
trp4Δ	YKO Collection	0.084	0.01324135
trp4Δ	This study	0.09225	0.01611159
met3Δ-trp4Δ	YKO Collection	0.68125	0.01099621
met3Δ-trp4Δ	This study	0.7315	0.03394604
trp2Δ	YKO Collection	0.1475	0.03276685
trp2Δ	This study	0.10675	0.0312023
trp4Δ	YKO Collection	0.078	0.00761577
trp4Δ	This study	0.08875	0.02482438
trp2Δ-trp4Δ	YKO Collection	0.79425	0.01314978
trp2Δ-trp4Δ	This study	0.80275	0.09768444
met14Δ	YKO Collection	0.118	0.01363818
met14Δ	This study	0.2515	0.02059935
arg2Δ	YKO Collection	0.1925	0.03837968
arg2Δ	This study	0.21775	0.03262284
met14Δ-arg2Δ	YKO Collection	0.55225	0.02232151
met14Δ-arg2Δ	This study	0.56725	0.02179258

Supplementary Table 4. Transition list of LC-MS assay to quantify anthranilate, tryptophan, and indole.

Molecule	Transition	Retention Time (min)	Fragmentor	Collision Energy
Anthranilic Acid	242.1>120	1.8+-0.5	54	4
Anthranilic Acid	242.1>105	1.8+-0.5	54	16
Tryptophan	309.1>263.1	1.2+-0.5	88	8
Tryptophan	309.1>105	1.2+-0.5	88	24
Indole	222.1>105.1	2.1±0.5	88	32
Indole	222.1>77.1	2.1±0.5	88	48

Supplementary Table 5. OD600 of *trp2*Δ, trp4Δ and controls in SM with anthranilate, tryptophan, and indole supplements.

Strain	Media	OD	Replicate	Mean_OD600	SD_OD600
WT	SM	1.528	R1	1.520333	0.006807
WT	SM	1.515	R2	1.520333	0.006807
WT	SM	1.518	R3	1.520333	0.006807
WT	SM+Trp	1.402	R1	1.44	0.052574
WT	SM+Trp	1.418	R2	1.44	0.052574
WT	SM+Trp	1.5	R3	1.44	0.052574
WT	SM+Ant	1.493	R1	1.498667	0.006028
WT	SM+Ant	1.498	R2	1.498667	0.006028
WT	SM+Ant	1.505	R3	1.498667	0.006028
trp2Δ+trp4Δ_1:1	SM	1.074	R1	1.085333	0.044106
trp2Δ+trp4Δ_1:1	SM	1.048	R2	1.085333	0.044106
trp2Δ+trp4Δ_1:1	SM	1.134	R3	1.085333	0.044106
trp2Δ+trp4Δ_1:2	SM	1.039	R1	1.024333	0.037233
trp2Δ+trp4Δ_1:2	SM	1.052	R2	1.024333	0.037233
trp2Δ+trp4Δ_1:2	SM	0.982	R3	1.024333	0.037233
trp2Δ+trp4Δ_1:5	SM	0.655	R1	0.641333	0.028113
trp2Δ+trp4Δ_1:5	SM	0.66	R2	0.641333	0.028113
trp2Δ+trp4Δ_1:5	SM	0.609	R3	0.641333	0.028113
trp2Δ+trp4Δ_2:1	SM	1.022	R1	0.950333	0.076957
trp2Δ+trp4Δ_2:1	SM	0.96	R2	0.950333	0.076957
trp2Δ+trp4Δ_2:1	SM	0.869	R3	0.950333	0.076957
trp2Δ+trp4Δ_5:1	SM	0.889	R1	0.687	0.17504
trp2Δ+trp4Δ_5:1	SM	0.592	R2	0.687	0.17504
trp2Δ+trp4Δ_5:1	SM	0.58	R3	0.687	0.17504
TRP2	SM	0.257	R1	0.257333	0.004509
TRP2	SM	0.262	R2	0.257333	0.004509
TRP2	SM	0.253	R3	0.257333	0.004509
TRP2	SM+Ant	1.333	R1	1.338333	0.005508
TRP2	SM+Ant	1.338	R2	1.338333	0.005508
TRP2	SM+Ant	1.344	R3	1.338333	0.005508
TRP2	SM+Trp	1.352	R1	1.356333	0.004041
TRP2	SM+Trp	1.36	R2	1.356333	0.004041
TRP2	SM+Trp	1.357	R3	1.356333	0.004041
TRP4	SM	0.369	R1	0.363333	0.016258
TRP4	SM	0.376	R2	0.363333	0.016258
TRP4	SM	0.345	R3	0.363333	0.016258

TRP4	SM+Ant	0.36	R1	0.359333	0.008021
TRP4	SM+Ant	0.351	R2	0.359333	0.008021
TRP4	SM+Ant	0.367	R3	0.359333	0.008021
TRP4	SM+Trp	1.541	R1	1.538333	0.009292
TRP4	SM+Trp	1.546	R2	1.538333	0.009292
TRP4	SM+Trp	1.528	R3	1.538333	0.009292

Supplementary Table 6. Script for pinning microbial arrays with the Singer-ROTOR (manual mode). A Singer-ROTOR was equipped with 96-density plating pads and was scripted to route liquid culture between source plates (a, b, x, y, z) and target plates (c1, c2, c3, n1, n2). A microbial library of 96 strains was distributed into output plates before test strains were overlaid. Output wells were pinned twice (receiving approximately 4 ul of liquid culture from source destinations). The 'Source Position' and 'Target Position' columns correspond to the 1:4 optional placement for pins when transferring liquid from 96- to 384-well microplates (see **Extended Data Figure 2** for more details). **Note:** Test strain plates (x, y, z) were duplicated (e.g,. x.1 and x.2) to avoid back-contamination of pins (i.e., avoiding "dirty" pins re-entering source wells, and thereby compromising future target destinations). The script's pinning itinerary was designed to consume minimal plating pads while generating target plate conditions.

Step	96-density Source	Source Position	384-density Target	Target Position
1	*Equip Clean Plating Pad*			
2	a	1	c1	1
3	a	1	c1	2
4	a	1	c2	1
5	a	1	c2	2
6	a	1	c3	1
7	a	1	c3	2
8	a	1	n1	1
9	a	1	n1	1
10	a	1	n1	2
11	a	1	n1	2
12	*Equip Clean Plating Pad*			
13	b	1	c1	3
14	b	1	c1	4
15	b	1	c2	3

16	b	1	c2	4
17	b	1	c3	3
18	b	1	c3	4
19	b	1	n2	1
20	b	1	n2	1
21	b	1	n2	2
22	b	1	n2	2
23	*Equip Clean Plating Pad*			
24	x.1	1	n1	3
25	x.1	1	n1	3
26	x.1	1	c1	1
27	x.1	1	c1	2
28	*Equip Clean Plating Pad*			
29	x.2	1	c1	3
30	x.2	1	c1	4
31	*Equip Clean Plating Pad*			
32	y.1	1	n1	4
33	y.1	1	n1	4
34	y.1	1	c2	1
35	y.1	1	c2	2
36	*Equip Clean Plating Pad*			
37	y.2	1	c2	3

38	y.2	1	c2	4
39	*Equip Clean Plating Pad*			
40	z.1	1	n2	3
41	z.1	1	n2	3
42	z.1	1	c3	1
43	z.1	1	c3	2
44	*Equip Clean Plating Pad*			
45	z.2	1	c3	3
46	z.2	1	c3	4

Supplementary Table 7. Oligonucleotide primers used in this work.

Purpose	Template	Oligo ID	Description	Sequence (5' – 3')
Amplification upstream **ARG2**	Amplification >100 bp upstream BY4741 - arg2Δ (gene disrupted with KanMX4 cassette)	oligoES23.2	Fw	GTCTTATTTCAGATCAGCCG
Amplification downstream **ARG2**	Amplification >100 bp downstream BY4741 - arg2Δ (gene disrupted with KanMX4 cassette)	oligoES23	Rv	GAACAAGTGTGCTATCTAGTGG
Amplification upstream **HIS2**	Amplification >100 bp upstream BY4741 - his2Δ (gene disrupted with KanMX4 cassette)	oligoLSV526	Fw	GCATGGGAGAGGTCTCCGGTTCGATTCCGGAC
Amplification downstream **HIS2**	Amplification >100 bp downstream BY4741 - his2Δ (gene disrupted with KanMX4 cassette)	oligoLSV527	Rv	CTCTTTCATGTTGAACTTCCAGATGCGGC
Amplification upstream **LYS12**	Amplification >100 bp upstream BY4741 - lys12Δ (gene disrupted with KanMX4 cassette)	oligoLSV536	Fw	CAAACGAGAACAACACATGTAGTGCTAAG
Amplification downstream **LYS12**	Amplification >100 bp downstream BY4741 - lys12Δ (gene disrupted with KanMX4 cassette)	oligoLSV537	Rv	GTAAAGATAAATCAAGACCACCACGTCCAC
Amplification upstream **MET1**	Amplification >100 bp upstream BY4741 - met1Δ (gene disrupted with KanMX4 cassette)	oligoLSV542	Fw	CTGTGAACGGACTCATAATGAAATTTGCTTC
Amplification downstream **MET1**	Amplification >100 bp downstream BY4741 - met1Δ (gene disrupted with KanMX4 cassette)	oligoLSV543	Rv	CAATTTACTCGAGATAAACTCTGTCCGTGC
Amplification upstream **MET3**	Amplification >100 bp upstream BY4741 - met3Δ (gene disrupted with KanMX4 cassette)	oligoES27.2	Fw	CGTAACAGTTGTGATATCG
Amplification downstream **MET3**	Amplification >100 bp downstream BY4741 - met3Δ (gene disrupted with KanMX4 cassette)	oligoES27	Rv	CCGTCTCCAAAAATTAACC
Amplification upstream **MET5**	Amplification >100 bp upstream BY4741 - met5Δ (gene disrupted with KanMX4 cassette)	oligoES28.2	Fw	GAGGCAGGAATGGTTTAAC
Amplification downstream **MET5**	Amplification >100 bp downstream BY4741 - met5Δ (gene disrupted with KanMX4 cassette)	oligoES28	Rv	GACACCGTAATTGCACATC
Amplification upstream **MET14**	Amplification >100 bp upstream BY4741 - met1Δ4 (gene disrupted with KanMX4 cassette)	oligoLSV548	Fw	GTGATGGTACGGCACCCACGGTACCTTAC
Amplification downstream **MET14**	Amplification >100 bp downstream BY4741 - met1Δ4 (gene disrupted with KanMX4 cassette)	oligoLSV549	Rv	CTTGAGCGCTCTGAGTATATTTAATGAATAATAG

Amplification upstream **TRP2**	Amplification >100 bp upstream BY4741 - trp2Δ (gene disrupted with KanMX4 cassette)	oligoES31.2	Fw	CGTTGAAGTAGTTTGTGGG
Amplification downstream **TRP2**	Amplification >100 bp downstream BY4741 - trp2Δ (gene disrupted with KanMX4 cassette)	oligoES31	Rv	CTTCCGTGAACAATACAAG
Amplification upstream **TRP4**	Amplification >100 bp upstream BY4741 - trp4Δ (gene disrupted with KanMX4 cassette)	oligoES32.2	Fw	CCACCTGCATTTTTCTTG
Amplification downstream **TRP4**	Amplification >100 bp downstream BY4741 - trp4Δ (gene disrupted with KanMX4 cassette)	oligoES32	Rv	GTAGGCGATACAACTTGTG
**KanMx4**	Colony PCR to verify insertion of the selection marker KanMx4	oligoLSV572	Fw	GCATGATGTGACTGTCGCCCGTACATTTAGCCC
**KanMx4**	Colony PCR to verify insertion of the selection marker KanMx4	oligoLSV573	Rv	GCACTTAACTTCGCATCTGGGCAGATGATGTCG
Sequencing	pWS041	oligoLSV604	Fw	GTCTCACTGAACTGGCC
Sequencing	pWS041	oligoLSV605	Rv	CGTGTATTGCACACATTTGTTTGG
Sequencing	pWS042	oligoLSV606	Fw	CCGACAGATCAAGGCAGTTAC
Sequencing	pWS042	oligoLSV607	Rv	CCACTGACGAGCAGATTTCC
Sequencing	pYTK096	oligoLSV610	Fw	CGTCGCAATACAACGCAGTTCGA
Sequencing	pYTK096	oligoLSV611	Rv	GGGGAGCGATTTGCAGGCATTTGCT

Supplementary Table 8. Plasmids used in this work.

Plasmid	Description	Antibiotic resistance	Source
**pYTK001**	Entry plasmid	CmR	John Dueber lab (Lee *et al*., 2015)
**pYTK096**	Integration vector, containing URA3	KanR	John Dueber lab (Lee *et al*., 2015)
**pYTK009**	Encondes for TDH3 promoter	CmR	John Dueber lab (Lee *et al*., 2015)
**pYTK053**	Encodes for tADH1 terminator	CmR	John Dueber lab (Lee *et al*., 2015)
**pWS041**	Pre-assembled vector for multicassette CDS-1	AmpR	Tom Ellis lab (Shaw *et al*., 2019)
**pWS042**	Pre-assembled vector for multicassette CDS-2	AmpR	Tom Ellis lab (Shaw *et al*., 2019)
**pWS043**	Pre-assembled vector for multicassette CDS-2	AmpR	Tom Ellis lab (Shaw *et al*., 2019)
**pWS044**	Pre-assembled vector for multicassette CDS-3	AmpR	Tom Ellis lab (Shaw *et al*., 2019)
**pWS064**	Integration vector, containing vLEU2	KanR	Tom Ellis lab (Shaw *et al*., 2019)
**pHP035**	pTDH3-mTagBFP2-tADH1-vLEU2	KanR	RLA laboratory (unpublished)
**pHP036**	pTDH3-mSCArlet-I-tADH1-vLEU2	KanR	RLA laboratory (unpublished)
**pHLUM v2**	Single-copy vector for auxotrophy compensation: HIS3, LEU2, URA3 and MET17	AmpR	Markus Ralser lab (Mulleder *et al*., 2016)
**pHLM**	Single-copy vector for auxotrophy compensation: HIS3, LEU2 and MET17	AmpR	Markus Ralser lab (Mulleder *et al*., 2016)
**pHUM**	Single-copy vector for auxotrophy compensation: HIS3, URA3 and MET17	AmpR	Markus Ralser lab (Mulleder *et al*., 2016)
**pLS207**	pYTK001 + TcPAND	CmR	This work
**pLS208**	pYTK001 + BcBAPAT	CmR	This work
**pLS226**	pWS41 + pTDH3 + BcBAPAT + tADH1	AmpR	This work
**pLS229**	pYTK096 + pTDH3 + TcPAND + tADH1	KanR	This work

Supplementary Table 9. Synthetic DNA sequences used in this work. All synthetic genes were codon-adapted suitably for expression in *Saccharomyces cerevisiae* and chemically synthesised (IDT or DNA2.0). Restriction sites are highlighted in green and blue and start and stop codons in bold.

Synthetic DNA	Sequence (5'- 3')	Restriction sites
Sequence encoding aspartate-1-decarboxylase (TcPAND) of *Tribolium castaneum*	GCATCGTCTCATCGGTCTCAT**ATG**CCTGCAACAGGAGAGGACCAAGA<br>CCTAGTTCAAGACTTAATAGAGGAACCAGCAACATTTAGTGATGCCGT<br>ATTATCCAGCGACGAGGAGCTATTTCATCAGAAGTGCCCTAAGCCTG<br>CTCCTATTTATAGCCCGGTGTCTAAACCCGTGTCCTTTGAATCATTGC<br>CTAACCGTAGGTTGCACGAGGAGTTTCTAAGGTCTTCTGTGGACGTTT<br>TATTACAGGAAGCTGTTTTCGAAGGTACGAACAGGAAGAACAGAGTC<br>CTTCAGTGGAGAGAGCCAGAGGAGCTAAGAAGGTTGATGGACTTCG<br>GAGTCAGGAGTGCCCCTTCTACCCACGAGGAGTTACTAGAGGTTTTA<br>AAGAAAGTAGTAACCTATAGTGTTAAAACAGGACACCCATATTTCGTT<br>AACCAACTATTTAGCGCGGTTGACCCTTATGGATTAGTTGCACAATGG<br>GCAACTGACGCTTTGAACCCTAGTGTATATACTTACGAGGTGAGTCCC<br>GTATTTGTATTGATGGAAGAGGTCGTCCTTAGGGAGATGAGGGCAAT<br>CGTCGGATTTGAGGGCGGTAAAGGGGACGGTATCTTCTGTCCTGGC<br>GGGTCAATAGCCAATGGCTACGCTATCTCCTGTGCAAGATATCGTTTT<br>ATGCCTGACATCAAGAAGAAGGGGTTGCATTCCTTGCCAAGATTAGTA<br>TTGTTCACATCTGAGGATGCGCACTATTCCATAAAGAAACTTGCATCT<br>TTCCAGGGCATTGGGACCGACAACGTATATTTGATACGTACCGACGC<br>AAGAGGCAGGATGGACGTTTCACACTTAGTTGAAGAGATAGAGCGTA<br>GCTTGAGGGAAGGGGCAGCACCTTTCATGGTTTCGGCTACCGCAGG<br>GACCACTGTTATCGGAGCTTTCGATCCTATAGAAAAGATCGCCGATGT<br>CTGCCAAAAGTACAAGCTTTGGCTACATGTAGACGCTGCGTGGGGTG<br>GCGGGGCATTGGTCAGCGCGAAGCACCGTCATCTACTGAAAGGAATT<br>GAGAGGGCTGATTCAGTTACATGGAACCCTCATAAGCTATTAACCGC<br>CCCACAGCAGTGCTCAACTTTATTATTGAGACATGAGGGTGTTCTAGC<br>TGAAGCACATTCCACTAATGCCGCTTACCTGTTCCAGAAGGACAAGTT<br>TTATGATACTAAGTACGACACAGGAGACAAACACATTCAGTGTGGAAG<br>GAGAGCAGACGTTCTAAAATTCTGGTTCATGTGGAAAGCAAAGGGCA<br>CAAGCGGGCTTGAGAAACACGTGGACAAAGTTTTCGAGAACGCCAGG<br>TTCTTTACAGATTGTATTAAGAACCGTGAGGGCTTTGAGATGGTAATT<br>GCAGAGCCAGAATACACAAACATATGTTTCTGGTATGTCCCAAAGTCT<br>CTACGTGGGCGTAAAGACGAGGCCGACTATAAAGACAAACTACACAA<br>AGTCGCTCCAAGAATCAAGGAGCGTATGATGAAAGAGGGCTCTATGA<br>TGGTAACCTATCAGGCACAGAAGGGTCACCCTAACTTCTTTCGTATAG<br>TCTTTCAAAACTCTGGACTAGACAAAGCCGACATGGTACATTTGGTCG<br>AAGAGATTGAGCGTCTAGGTTCTGATTTA**TGA**ATCCTGAGACCTGAGA<br>CGGCAT	*BsmbI* / *BsaI*
Sequence encoding β-alanine-pyruvate aminotransferase (BcBAPAT) of *Bacillus cereus*	GCATCGTCTCATCGGTCTCAT**ATG**GAATTAATGATCGTTCAAGTAACT<br>GAGCAAACACAGAGCTTAAAGAAGACCGACGAGAAATATTTATGGCA<br>TGCCATGAGGGGCGCAGCGCCATCTCCGACTAATTTGATCATTACCA<br>AAGCAGAGGGTGCGTGGGTCACCGATATTGATGGTAATCGTTATCTA<br>GATGGCATGTCTGGCTTGTGGTGTGTTAACGTCGGCTATGGGAGAAA<br>AGAACTAGCAAGGGCTGCATTCGAACAACTTGAAGAAATGCCTTACTT<br>CCCTTTAACTCAGTCTCATGTACCTGCAATAAAGCTAGCAGAGAAATT<br>GAATGAGTGGCTGGATGACGAATACGTCATTTTCTTCTCCAACTCTGG<br>GTCGGAAGCTAATGAAACGGCATTCAAAATAGCAAGACAATATCACCA<br>ACAGAAAGGTGACCACGGTAGATATAAGTTTATTTCAAGATATCGTGC<br>GTATCATGGAAATTCCATGGGTGCCCTGGCTGCAACTGGTCAAGCCC<br>AAAGAAAATACAAGTACGAGCCACTAGGGCAAGGATTCCTACACGTA<br>GCCCCACCTGATACCTACAGAAATCCAGAAGATGTCCATACCCTGGC<br>ATCAGCTGAGGAAATCGATCGTGTTATGACGTGGGAATTGTCTCAAAC<br>CGTGGCTGGAGTGATCATGGAACCCATCATTACGGGTGGTGGTATTC<br>TGATGCCACCTGATGGATATATGGAAAAGGTAAAGGAAATTTGCGAAA<br>AGCACGGAGCACTTTTAATCTGCGATGAGGTTATCTGCGGATTTGGC	*BsmbI* / *BsaI*

	CGTACTGGGAAACCGTTTGGGTTCATGAACTATGGTGTCAAGCCGGA CATTATTACTATGGCGAAAGGTATCACCAGCGCATACCTACCTCTTTC TGCCACAGCTGTGCGTCGTGAAGTGTACGAGGCTTTTGTAGGTTCCG ACGATTACGACAGATTTAGACATGTAAACACCTTTGGTGGAAATCCAG CAGCCTGTGCATTGGCTTTAAAGAACCTGGAAATAATGGAAAACGAAA AGCTGATTGAAAGATCAAAGGAACTGGGTGAAAGATTGTTGTACGAAT TAGAGGATGTGAAGGAACATCCCAATGTGGGTGATGTAAGGGGCAAA GGTTTGTTGCTGGGAATCGAACTAGTTGAGGATAAACAAACTAAGGAA CCTGCATCTATAGAAAAGATGAATAAGGTTATTAATGCATGCAAGGAG AAAGGACTGATCATCGGGAAGAATGGGGATACAGTAGCTGGGTATAA CAATATCCTACAACTGGCGCCCCCGTTGTCAATCACCGAAGAGGACT TCACTTTTATAGTTAAAACGATGAAGGAATGTCTTGCTCAACTA**TGA** ATCCTGAGACCTGAGACGGCAT	

Supplementary Table 10. Description of *S. cerevisiae* strains used in this work.

Strain	Deleted ORF	Parental strain	Description	Source
**BY4741**	**MATa**, his3Δ, leu2Δ, met15Δ, ura3Δ		Wild-type strain	RLA laboratory
**BY4741-*TcPAND-BcBAPAT***	MATa his3Δ, leu2Δ, met15Δ, ura3Δ	BY4741	pTDH3-TcPAND-tADH1+ pTDH3-BcBAPAT-tADH1+ pHLM	RLA laboratory
arg2Δ **(YKO)**	**arg2Δ**, MATa, his3Δ, leu2Δ, met15Δ, ura3Δ	BY4741	Obtained from YKO collection	YKO Collection
arg2Δ	**arg2Δ**, MATa, his3Δ, leu2Δ, met15Δ, ura3Δ	BY4741	Re-construction of arg2Δ, gene disrupted with kanMx4	RLA laboratory
arg2Δ-BFP	**arg2Δ**, MATa, his3Δ, leu2Δ, met15Δ, ura3Δ	BY4741	mTAG-BFP-Leu + pHUM	RLA laboratory
his2Δ **(YKO)**	**his1Δ**, MATa, his3Δ, leu2Δ, met15Δ, ura3Δ	BY4741	Obtained from YKO collection	YKO Collection
his2Δ	**his2Δ**, MATa, his3Δ, leu2Δ, met15Δ, ura3Δ	BY4741	Re-construction of his2Δ, gene disrupted with kanMx4	RLA laboratory
his2Δ-BFP	**his2Δ**, MATa, his3Δ, leu2Δ, met15Δ, ura3Δ	BY4741	mTAG-BFP-Leu + pHUM	RLA laboratory
his2Δ-TcPAND	**his2Δ**, MATa, his3Δ, leu2Δ, met15Δ, ura3Δ	BY4741	pTDH3-TcPAND-tADH1 + pHLM	RLA laboratory
his2Δ-BcBAPAT	**his2Δ**, MATa, his3Δ, leu2Δ, met15Δ, ura3Δ	BY4741	pTDH3-BcBAPAT -tADH1+ pHLM	RLA laboratory
lys12Δ **(YKO)**	**lys12Δ**, MATa, his3Δ, leu2Δ, met15Δ, ura3Δ	BY4741	Obtained from YKO collection	YKO Collection
lys12Δ	**lys12Δ**, MATa, his3Δ, leu2Δ, met15Δ, ura3Δ	BY4741	Re-construction of lys12Δ, gene disrupted with kanMx4	RLA laboratory
lys12Δ-mScarlet	**lys12Δ**, MATa, his3Δ, leu2Δ, met15Δ, ura3Δ	BY4741	mScarlet-I-Leu + pHUM	RLA laboratory
met1Δ **(YKO)**	**met1Δ**, MATa, his3Δ, leu2Δ, met15Δ, ura3Δ	BY4741	Obtained from YKO collection	YKO Collection
met1Δ	**met1Δ**, MATa, his3Δ, leu2Δ, met15Δ, ura3Δ	BY4741	Re-construction of met1Δ, gene disrupted with kanMx4	RLA laboratory

met1Δ-BFP	**met1Δ**, MATa, his3Δ, leu2Δ, met15Δ, ura3Δ	BY4741	mTAG-BFP-Leu + pHUM	RLA laboratory
met1Δ-mScarlet	**met1Δ**, MATa, his3Δ, leu2Δ, met15Δ, ura3Δ	BY4741	mScarlet-I-Leu + pHUM	RLA laboratory
met3Δ **(YKO)**	**met3Δ**, MATa, his3Δ, leu2Δ, met15Δ, ura3Δ	BY4741	Obtained from YKO collection	YKO Collection
met3Δ	**met3Δ**, MATa, his3Δ, leu2Δ, met15Δ, ura3Δ	BY4741	Re-construction of met3Δ, gene disrupted with kanMx4	RLA laboratory
met3Δ-mScarlet	**met3Δ**, MATa, his3Δ, leu2Δ, met15Δ, ura3Δ	BY4741	mScarlet-I-Leu + pHUM	RLA laboratory
met3Δ-TcPAND	**met3Δ**, MATa, his3Δ, leu2Δ, met15Δ, ura3Δ	BY4741	pTDH3-TcPAND-tADH1 + pHLM	RLA laboratory
met3Δ-BcBAPAT	**met3Δ**, MATa, his3Δ, leu2Δ, met15Δ, ura3Δ	BY4741	pTDH3-BcBAPAT -tADH1+ pHLM	RLA laboratory
met5Δ **(YKO)**	**met15Δ**, MATa, his3Δ, leu2Δ, met15Δ, ura3Δ	BY4741	Obtained from YKO collection	YKO Collection
met5Δ	**met5Δ**, MATa, his3Δ, leu2Δ, met15Δ, ura3Δ	BY4741	Re-construction of met5Δ, gene disrupted with kanMx4	RLA laboratory
met5Δ-BFP	**met5Δ**, MATa, his3Δ, leu2Δ, met15Δ, ura3Δ	BY4741	mTAG-BFP-Leu + pHUM	RLA laboratory
met5Δ-mScarlet	**met5Δ**, MATa, his3Δ, leu2Δ, met15Δ, ura3Δ	BY4741	mScarlet-I-Leu + pHUM	RLA laboratory
met14Δ **(YKO)**	**met14Δ**, MATa, his3Δ, leu2Δ, met15Δ, ura3Δ	BY4741	Obtained from YKO collection	YKO Collection
met14Δ	**met14Δ**, MATa, his3Δ, leu2Δ, met15Δ, ura3Δ	BY4741	Re-construction of met1Δ4, gene disrupted with kanMx4	RLA laboratory
met14Δ-mScarlet	**met14Δ**, MATa, his3Δ, leu2Δ, met15Δ, ura3Δ	BY4741	mScarlet-I-Leu + pHUM	RLA laboratory
met14Δ-TcPAND	**met14Δ**, MATa, his3Δ, leu2Δ, met15Δ, ura3Δ	BY4741	pTDH3-TcPAND-tADH1 + pHLM	RLA laboratory
met14Δ-BcBAPAT	**met14Δ**, MATa, his3Δ, leu2Δ, met15Δ, ura3Δ	BY4741	pTDH3-BcBAPAT -tADH1+ pHLM	RLA laboratory
trp2Δ **(YKO)**	**trp2Δ**, MATa, his3Δ, leu2Δ, met15Δ, ura3Δ	BY4741	Obtained from YKO collection	YKO Collection

trp2Δ	**trp2Δ**, MATa, his3Δ, leu2Δ, met15Δ, ura3Δ	BY4741	Re-construction of trp2Δ, gene disrupted with kanMx4	RLA laboratory
trp2Δ-mScarlet	**trp2Δ**, MATa, his3Δ, leu2Δ, met15Δ, ura3Δ	BY4741	mScarlet-I-Leu + pHUM	RLA laboratory
trp2Δ-TcPAND	**trp2Δ**, MATa, his3Δ, leu2Δ, met15Δ, ura3Δ	BY4741	pTDH3-TcPAND-tADH1 + pHLM	RLA laboratory
trp2Δ-BcBAPAT	**trp2Δ**, MATa, his3Δ, leu2Δ, met15Δ, ura3Δ	BY4741	pTDH3-BcBAPAT -tADH1+ pHLM	RLA laboratory
trp4Δ (YKO)	**trp4Δ**, MATa, his3Δ, leu2Δ, met15Δ, ura3Δ	BY4741	Obtained from YKO collection	YKO Collection
trp4Δ	**trp4Δ**, MATa, his3Δ, leu2Δ, met15Δ, ura3Δ	BY4741	Re-construction of trp4Δ, gene disrupted with kanMx4	RLA laboratory
trp4Δ-BFP	**trp4Δ**, MATa, his3Δ, leu2Δ, met15Δ, ura3Δ	BY4741	mTAG-BFP-Leu + pHUM	RLA laboratory
trp4Δ-TcPAND	**trp4Δ**, MATa, his3Δ, leu2Δ, met15Δ, ura3Δ	BY4741	pTDH3-TcPAND-tADH1 + pHLM	RLA laboratory
trp4Δ-BcBAPAT	**trp4Δ**, MATa, his3Δ, leu2Δ, met15Δ, ura3Δ	BY4741	pTDH3-BcBAPAT -tADH1+ pHLM	RLA laboratory
