# Supplementary material for: Spontaneously established syntrophic yeast communities improve bioproduction
Source: Nat Chem Biol. 2023 May 29;19(8):951–61. doi: 10.1038/s41589-023-01341-2 (PMC10374442; doi:10.1038/s41589-023-01341-2)
Supplement: Supplementary file 2 — Reporting Summary [file 41589_2023_1341_MOESM2_ESM.pdf]

nature portfolio

Corresponding author(s): Rodrigo Ledesma-Amaro

Last updated by author(s): Mar 13, 2023

Reporting Summary

Nature Portfolio wishes to improve the reproducibility of the work that we publish. This form provides structure for consistency and transparency in reporting. For further information on Nature Portfolio policies, see our Editorial Policies and the Editorial Policy Checklist.

Statistics

For all statistical analyses, confirm that the following items are present in the figure legend, table legend, main text, or Methods section.

n/a	Confirmed	
☐	☒	The exact sample size (*n*) for each experimental group/condition, given as a discrete number and unit of measurement
☐	☒	A statement on whether measurements were taken from distinct samples or whether the same sample was measured repeatedly
☐	☒	The statistical test(s) used AND whether they are one- or two-sided<br>*Only common tests should be described solely by name; describe more complex techniques in the Methods section.*
☒	☐	A description of all covariates tested
☐	☒	A description of any assumptions or corrections, such as tests of normality and adjustment for multiple comparisons
☐	☒	A full description of the statistical parameters including central tendency (e.g. means) or other basic estimates (e.g. regression coefficient) AND variation (e.g. standard deviation) or associated estimates of uncertainty (e.g. confidence intervals)
☐	☒	For null hypothesis testing, the test statistic (e.g. *F*, *t*, *r*) with confidence intervals, effect sizes, degrees of freedom and *P* value noted<br>*Give P values as exact values whenever suitable.*
☒	☐	For Bayesian analysis, information on the choice of priors and Markov chain Monte Carlo settings
☒	☐	For hierarchical and complex designs, identification of the appropriate level for tests and full reporting of outcomes
☒	☐	Estimates of effect sizes (e.g. Cohen's *d*, Pearson's *r*), indicating how they were calculated

Our web collection on statistics for biologists contains articles on many of the points above.

Software and code

Policy information about availability of computer code

Data collection	Scripting operations related to data cleaning & processing from instrumental outputs were performed in R Studio (v3.12) and written in the R programming language. The scripts themselves can be found in the Supplemental Section. Required packages included: tidyverse, grid, gridExtra, stringr, xlsx, reshape2, ggrepel, data.table, userfriendlyscience, and gtools. Information regarding each package's use can be found within the CRAN Repository. High throughput colony processing was achieved with the RoToR HDA pinning robot (Singer Instruments).
Data analysis	All R code used for the analysis can be found in Supplementary Code on GitHub: https://github.com/Ralser-lab/auxcocul

For manuscripts utilizing custom algorithms or software that are central to the research but not yet described in published literature, software must be made available to editors and reviewers. We strongly encourage code deposition in a community repository (e.g. GitHub). See the Nature Portfolio guidelines for submitting code & software for further information.

Data

Policy information about availability of data

All manuscripts must include a data availability statement. This statement should provide the following information, where applicable:

- Accession codes, unique identifiers, or web links for publicly available datasets
- A description of any restrictions on data availability
- For clinical datasets or third party data, please ensure that the statement adheres to our policy

All plate reader data generated or analysed during the presented co-culturing screen are included in this published article (and its supplementary information files). Additional datasets generated during and/or analysed during the current study are available from the corresponding author upon request.

Human research participants

Policy information about studies involving human research participants and Sex and Gender in Research.

Reporting on sex and gender	Not applicable
Population characteristics	Not applicable
Recruitment	Not applicable
Ethics oversight	Not applicable

Note that full information on the approval of the study protocol must also be provided in the manuscript.

Field-specific reporting

Please select the one below that is the best fit for your research. If you are not sure, read the appropriate sections before making your selection.

☒ Life sciences ☐ Behavioural & social sciences ☐ Ecological, evolutionary & environmental sciences

For a reference copy of the document with all sections, see nature.com/documents/nr-reporting-summary-flat.pdf

Life sciences study design

All studies must disclose on these points even when the disclosure is negative.

Sample size	Whole genome screen. Strains tested were chosen based on growth in minimal media and is well described in the text. Samples sizes were maximized and limited by the amount of wells available across 96- and 384-well microtiter plates while maintaining appropriate controls.
Data exclusions	Quality control filters that were applied to the raw data are clearly described in the text.
Replication	4 or 92 replicates (separate cultures in a multi-well plate) of each monoculture and co-culture pair. All attempts at replication were successful.
Randomization	Replicates of each monoculture - coculture test groups were positioned randomly in different areas of a plate and across plates.
Blinding	Not applicable. All experiments were carried out using a pipetting robot and all data was analyzed in R. Therefore, no subjective analysis from the person conducting the experiment was involved.

Reporting for specific materials, systems and methods

We require information from authors about some types of materials, experimental systems and methods used in many studies. Here, indicate whether each material, system or method listed is relevant to your study. If you are not sure if a list item applies to your research, read the appropriate section before selecting a response.

Materials & experimental systems

n/a	Involved in the study
☒	☐ Antibodies
☒	☐ Eukaryotic cell lines
☒	☐ Palaeontology and archaeology
☒	☐ Animals and other organisms
☒	☐ Clinical data
☒	☐ Dual use research of concern

Methods

n/a	Involved in the study
☒	☐ ChIP-seq
☒	☐ Flow cytometry
☒	☐ MRI-based neuroimaging
